# Alterations of Mitochondrial Structure in Methamphetamine Toxicity

**DOI:** 10.3390/ijms23168926

**Published:** 2022-08-10

**Authors:** Paola Lenzi, Francesca Biagioni, Carla L. Busceti, Gloria Lazzeri, Maico Polzella, Alessandro Frati, Michela Ferrucci, Francesco Fornai

**Affiliations:** 1 Department of Translational Research and New Technologies in Medicine and Surgery, University of Pisa, Via Roma 55, 56126 Pisa, Italy; 2 Istituto di Ricovero e Cura a Carattere Scientifico (I.R.C.C.S.) Neuromed, Via Atinense 18, 86077 Pozzilli, Italy; 3 Aliveda Laboratories, Viale Karol Wojtyla, 19, 56042 Crespina Lorenzana, Italy; 4 Neurosurgery Division, Department of Human Neurosciences, Sapienza University, 00135 Roma, Italy

**Keywords:** neurotoxicity, psychostimulants, MitoTracker, ultrastructural morphometry, mitochondrial fission, mitophagy, Fis1, DRP1, Pink1, Parkin

## Abstract

Recent evidence shows that methamphetamine (METH) produces mitochondrial alterations that contribute to neurotoxicity. Nonetheless, most of these studies focus on mitochondrial activity, whereas mitochondrial morphology remains poorly investigated. In fact, morphological evidence about the fine structure of mitochondria during METH toxicity is not available. Thus, in the present study we analyzed dose-dependent mitochondrial structural alterations during METH exposure. Light and transmission electron microscopy were used, along with ultrastructural stoichiometry of catecholamine cells following various doses of METH. In the first part of the study cell death and cell degeneration were assessed and they were correlated with mitochondrial alterations observed using light microscopy. In the second part of the study, ultrastructural evidence of specific mitochondrial alterations of crests, inner and outer membranes and matrix were quantified, along with in situ alterations of mitochondrial proteins. Neurodegeneration induced by METH correlates significantly with specific mitochondrial damage, which allows definition of a scoring system for mitochondrial integrity. In turn, mitochondrial alterations are concomitant with a decrease in fission/mitophagy protein Fis1 and DRP1 and an increase in Pink1 and Parkin in situ, at the mitochondrial level. These findings provide structural evidence that mitochondria represent both direct and indirect targets of METH-induced toxicity.

## 1. Introduction

Methamphetamine (METH) is a psychostimulant that is neurotoxic for monoamine neurons and most dopamine (DA)-containing neurons [1,2,3,4,5]. The selective toxicity of METH is based on the selective uptake by presynaptic transporters, namely the DA transporter (DAT) [6], norepinephrine transporter (NET) and serotonin transporter (SERT) [7]. Nonetheless, some passive diffusion of METH also occurs [8], which explains toxicity beyond catecholamine cells. The binding of METH to plasma membrane catecholamine transporters, apart from promoting the entry of METH into the neuron, promotes catecholamine release. In fact, METH reverts the direction of transporter activity, which is switched to extrude catecholamines from the neuron to the extracellular space [6,9]. Once METH is in the neuron, selective toxicity occurs due to the binding of METH to catecholamine-containing vesicles, where it blocks and translocates vesicular monoamine transporter type-2 (VMAT-2) [10,11]. At this level, METH also disrupts the vesicular protonic pump. In this way, vesicular DA is no longer a polar compound and freely spreads out of the vesicle [12,13,14]. Thus, a massive DA efflux into the cytosol takes place [15,16]. Increased DA levels are metabolized by monoamine oxidase (MAO) [17,18]. Therefore, a massive amount of DA undergoes oxidation [19,20] and it produces reactive oxidative species (ROS) that mis-fold protein structures, and may extend oxidation to mitochondria, which are functionally impaired [19,21,22,23,24]. In fact, METH-induced oxidative stress is critical for METH-induced neurotoxicity [25,26,27,28].

In fact, a recent study indicates that within mitochondrial MAO, DA is metabolized into toxic oxidative species [29], thus leading to mitochondrial stress, which along with direct and indirect effects such as endoplasmic reticulum stress, calcium release, DNA instability and protein oxidation [30], may sustain mitochondrial damage.

In line with this, growing evidence shows that mitochondrial function is affected at multiple levels of the respiratory chain following METH exposure [31]. In fact, the role of mitochondria in METH-induced toxicity has been emphasized recently [31,32,33]. Recent evidence shows that METH produces a variety of mitochondrial alterations that contribute to neurotoxicity and behavioral sensitization [34]. Nonetheless, most studies focused on mitochondrial activity, whereas mitochondrial morphology remains poorly investigated. In detail, the ultrastructure of mitochondria was never analyzed to substantiate the fine morphological changes following METH exposure. Thus, in the present study we address the morphological aspects of mitochondrial alterations under the effects of METH. Light (both MitoTracker-Red, MTR-R, and MitoTracker-Green, MTR-G) and transmission electron microscopy (TEM, both plain ultrastructural morphometry and immunogold stoichiometry) are used to investigate the type of deleterious effects produced by METH on mitochondria. For this aim, catecholamine cells, which are the natural targets of METH, were exposed to various doses of METH. In detail, PC12 cells were used due to their well-characterized cell profile, which we investigated in a previous study [35]. When considering the biochemistry and morphometry of PC12 cells, although some discrepancies exist, they are quite similar to catecholamine terminals [35]. In the first part of the study cell death (quantified by cell loss) and cell degeneration (quantified by persistent derangement of spared cells) were correlated with mitochondrial alterations detected by light microscopy.

In detail, we measured cell death (i.e., cells with complete loss of viability) by light microscopy, by hematoxylin and eosin (H&E) staining, which allows indirect measurement of cell death by detecting the occurrence of spared cells (since no staining can be produced by lost cells). In contrast, cell degeneration, which indicates the occurrence of altered though still viable cells, can be visualized through different staining procedures. These include trypan blue (TB) and Fluoro-Jade B (FJB) that reveal dye-specific cell damage (i.e., loss of membrane integrity for TB [36], expression of specific proteins that are produced by dying neurons for FJB [37]).

In the second part of the study, ultrastructural evidence of specific mitochondrial alterations and in situ alterations of mitochondrial proteins, which are key to regulating mitochondrial turn over, were investigated. These include key proteins for mitochondrial fission and mitophagy such as mitochondrial fission 1 protein (Fis1), dynamin-related protein 1 (DRP1), PTEN-induced putative kinase1 (Pink) and Parkin.

## 2. Results

### 2.1. Dose-Dependent Effects of METh on Cell Survival and Cell Degeneration

**Figure 1:**
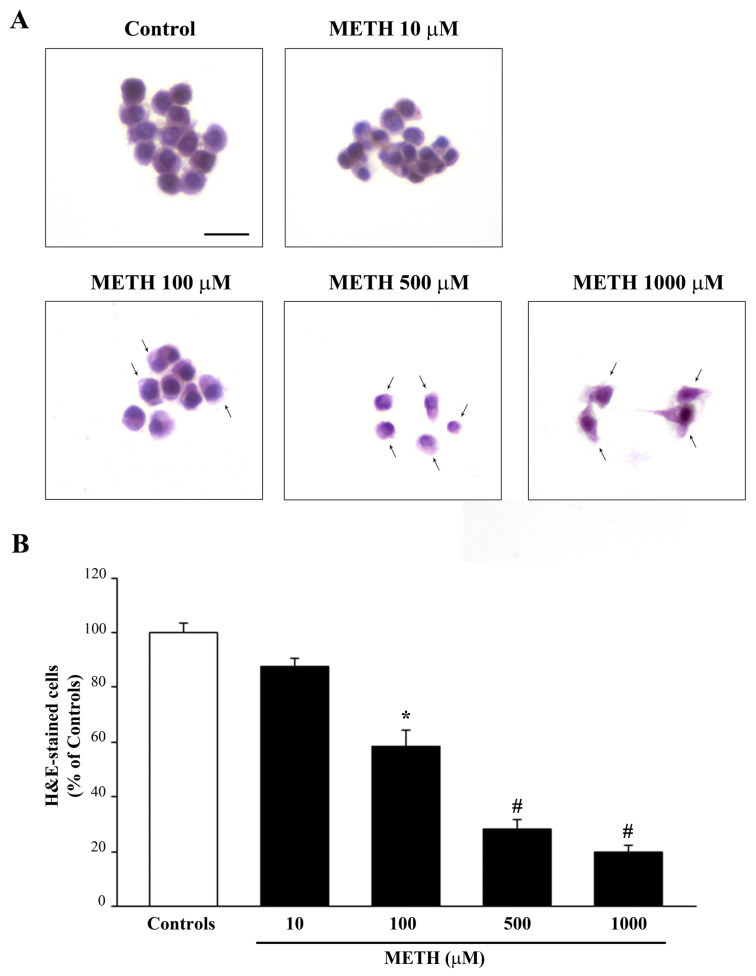
METH dose-dependently induces cell death. (**A**) Representative pictures of H&E-stained cells show that increasing doses of METH (from 10 μM up to 1000 μM) dose-dependently induces cell loss and morphological changes in spared cells (arrows). (**B**) The graph reports the percentage of cells counted after METH treatment (at doses ranging from 10 μM up to 1000 μM) compared with those counted in control conditions. Values are given as the mean percentage ± S.E.M. of cells counted from three independent experiments (assuming controls as 100%). * *p* ≤ 0.05 compared with controls; # *p* ≤ 0.05 compared with controls and METH 100 μM. Scale bar = 14 μm.

As shown in the representative pictures of Figure 1A, METH dose-dependently reduces the amount of catecholamine cells. In these experimental conditions, cell loss occurs significantly, starting at a dose of METH 100 μM and it increases up to a dose 1 mM, which produce a loss of catecholamine cells surpassing 80% (graph of Figure 1B). The occurrence of cell death is calculated by light microscopy by plain H&E staining.

**Figure 2:**
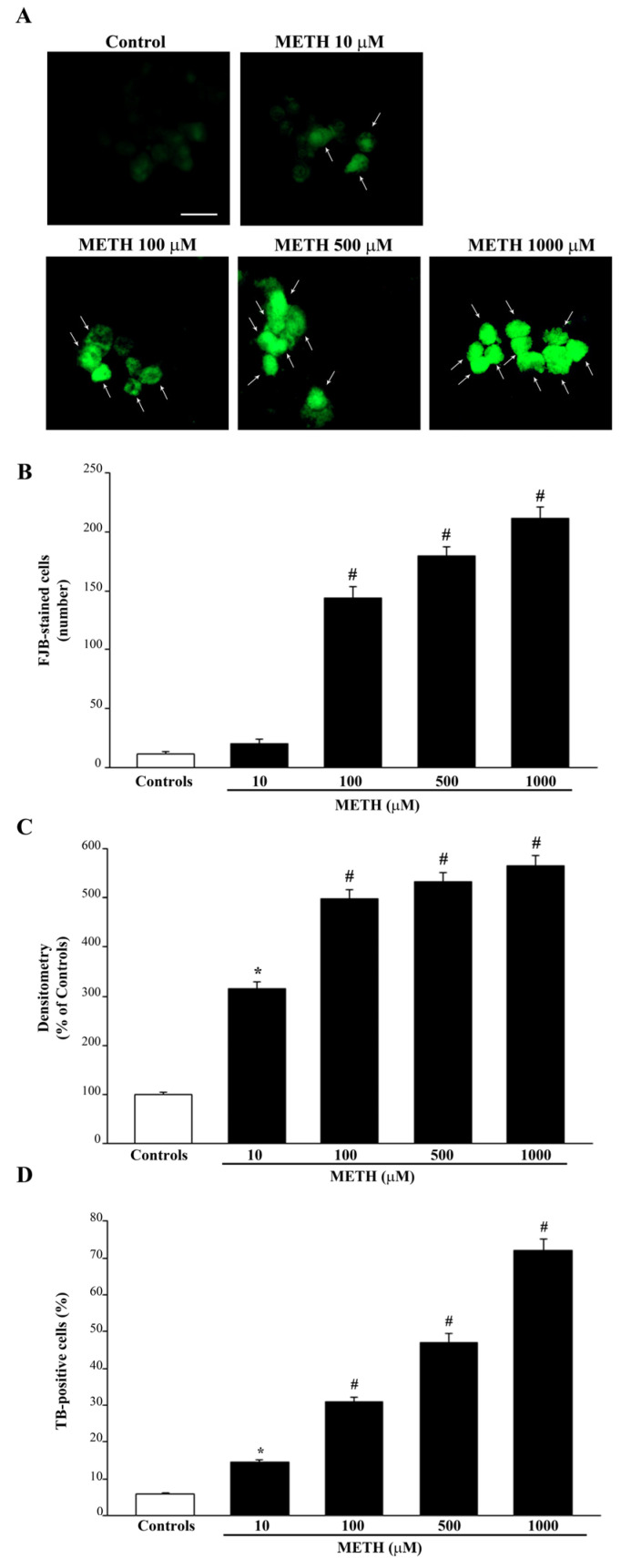
METH dose-dependently increases FJB histofluorescence and TB-positive cells. (**A**) Representative pictures of FJB-stained PC12 cells after treatment with increasing doses (from 10 μM up to 1000 μM) of METH show a dose-dependent increase in FJB histofluorescence induced by METH compared with controls. Arrows indicate FJB intensely positive cells. This is confirmed by the graphs shown in (**B**,**C**), which report the number of FJB-positive cells and the mean intensity of fluorescence per cell, respectively, both in control conditions and following increasing doses of METH. (**D**) The graph reports the count of TB-positive cells following increasing doses of METH (from 10 μM up to 1000 μM) compared with controls. Values are given as the mean ± S.E.M (**B**) the mean percentage ± S.E.M. (assuming controls as 100%, (**C**)) or the mean percentage ± S.E.M. of TB-positive cells out of the total cells (**D**), which were counted from three independent experiments. * *p* ≤ 0.05 compared with controls; # *p* ≤ 0.05 compared with controls and METH 10 μM. Scale bar = 14 μm.

When METH-induced cell degeneration was assessed, Fluoro-Jade B (FJB) histofluorescence was used along with the marker for dying cells, Trypan Blue (TB). As expected, following FJB staining cell degeneration was evident for doses of METH lower than those required to produce cell death. In fact, FJB-positive cells were significantly detected even at the lowest dose of METH, 10 μM, as shown in Figure 2. As shown in representative Figure 2A and reported in the graph of Figure 2B, the number of FJB-positive cells further increases following METH 100 μM, up to 500 μM and 1000 μM (Figure 2C). Similarly, the number of TB-positive cells is increased compared with control even at the 10 μM dose of METH (Figure 2D). When METH is administered at higher doses there is a concomitant increase in cell degeneration and occurrence of significant cell loss as measured in the graphs of Figure 1B and Figure 2B–D.

It is remarkable that, depending on the method, the dose response for increasing doses of METH shows slight variations. In fact, when considering FJB histofluorescence the plateau for cell degeneration is already present at the dose 100 μM of METH. In contrast, when measuring cell loss there is a significant further increase of cell death when increasing the dose of METH from 100 μM up to 1000 μM (Figure 1B). Similarly, a significant increase of degeneration is detected when comparing 100 μM and 1000 μM when counting the number (not the intensity) of FJB-positive cells (Figure 2B) or when counting the number of positive cells detected by TB staining (Figure 2D).

### 2.2. Dose-Dependent Effects of METH on the Amount of Total Mitochondria (Mitotracker Green, MTR-G)

**Figure 3:**
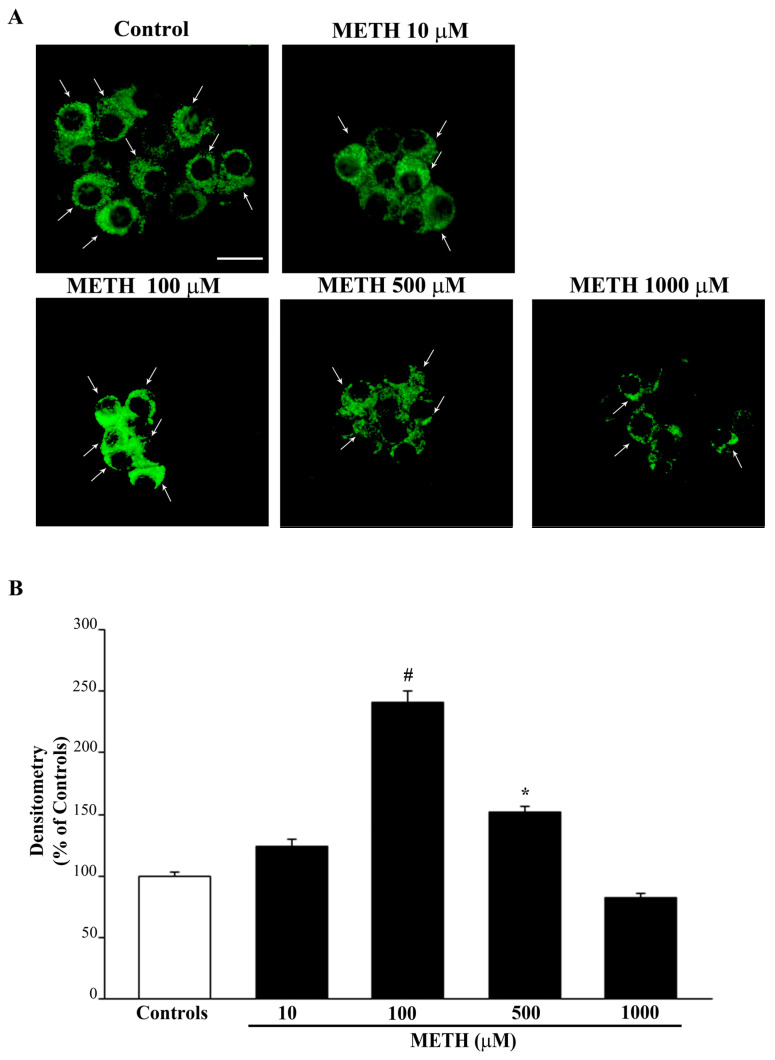
Effect of METH on MTR-G fluorescence. (**A**) Representative pictures of MTR-G fluorescence, which labels total (both healthy and altered) mitochondria, show that MTR-G-positive mitochondria increase at the dose of METH 100 μM, whereas they are similar to controls following higher METH doses (i.e., 1000 μM). Arrows indicate intensely MTR-G-stained cells. This is confirmed by the graph (**B**), which reports the densitometry of MTR-G fluorescence. Values are given as the mean percentage ± S.E.M. of optical density (assuming controls as 100%) from three independent experiments. * *p* < 0.05 compared with controls; # *p* < 0.05 compared with all other groups. Scale bar = 11 μm.

When staining with MTR-G was carried out, the amount of total mitochondria were increased by moderate doses of METH (peak effect at the dose of 100 μM as shown in representative Figure 3A and graph of Figure 3B). MTR-G stains both healthy and damaged mitochondria, thus leaving the interpretation of these data dependent on the mitochondrial status.

**Figure 4:**
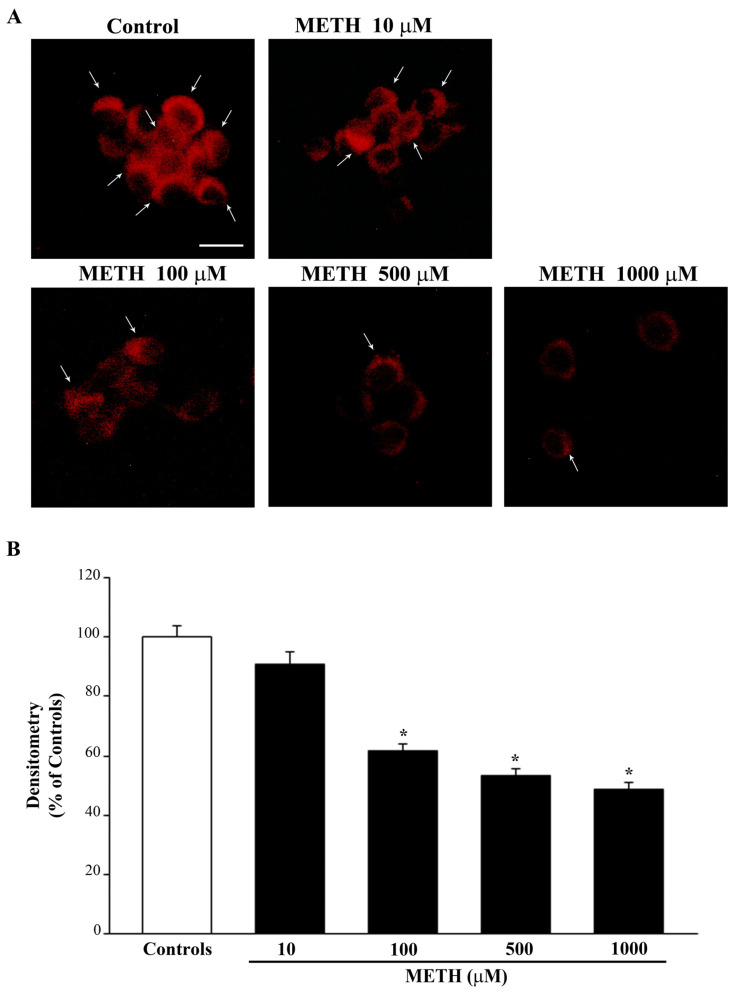
METH dose-dependently reduces MTR-R fluorescence. MTR-R labels healthy mitochondria. (**A**) Representative pictures showing a dose-dependent decrease of MTR-R fluorescence for doses starting at METH 100 μM. Arrows indicate intensely MTR-R-stained cells. (**B**) The graph reports the mean densitometry of MTR-R fluorescence per cell. Values are given as the mean percentage ± S.E.M. of optical density (assuming controls as 100%) from three independent experiments. * *p* < 0.05 compared with controls. Scale bar = 11 μm.

When staining the cells with MTR-R, it is evident that METH dose-dependently decreases the number of healthy mitochondria (representative Figure 4A and graph of Figure 4B). It is remarkable that the 100 μM dose of METH produces the maximum loss of healthy mitochondria, which remains steady for doses of METH up to 1000 μM (Figure 4B). These effects quite overlap with the dose-response for neurodegeneration as detected by the number of FJB-positive cells and intensity of FJB per cell (Figure 2B,C, respectively).

Thus, the decrease in healthy mitochondria detected by MTR-R seems to be a reliable marker for METH-induced neurodegeneration, whereas it does not provide a reliable index of METH-induced cell death. In fact, although cell death further increases for doses above METH 100 μM, MTR-R staining reaches the maximum decrease at METH 100 μM, which corresponds to the dose producing the maximum FJB intensity per cell. Data obtained combining MTR-R staining with MTR-G staining allow us to infer which kind of changes are produced by METH in the mitochondrial compartment. In fact, assuming that the dose of METH 100 μM reaches a plateau to induce mitochondrial damage (as shown by the maximum decrease of MTR-R, Figure 4B), it is likely that the increase in MTR-G (staining all mitochondria) is mostly due to damaged mitochondria that are not removed from the cell. This number decreases when cell death occurs. In fact, light microscopy shows that METH reduces the number of healthy mitochondria despite increasing the number of total mitochondria when cells keep their structural integrity, which is dramatically lost for METH doses above 100 μM. However, these interpretations suffer the limits inherent to indirect mitochondrial staining techniques. Further explanation of these effects require a finer and direct approach, which is provided by ultrastructural morphometry.

### 2.3. Dose-Dependent Effects of METH on TEM-Detected Cell Death Correlates with Mitochondral Alterations

**Figure 5:**
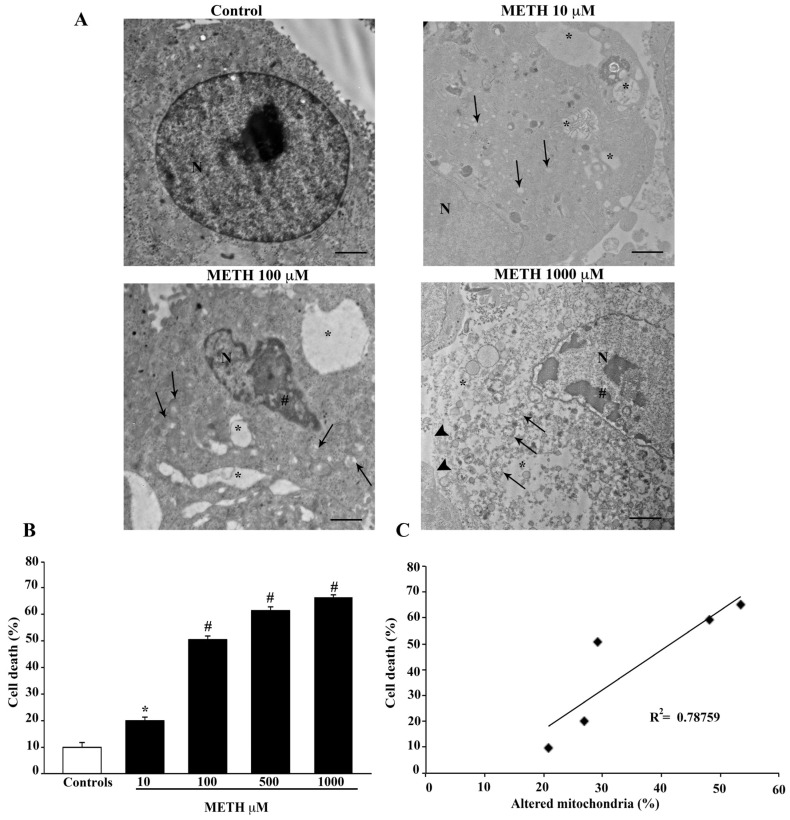
METH-induced cell death correlates with mitochondrial alterations. (**A**) Representative TEM micrographs showing healthy (Control) and damaged cells following increasing doses (10 μM, 100 μM, 1000 μM) of METH. After METH treatment, nuclear condensation (#), large cytosolic vacuoles (*), altered mitochondria (arrows) and fragmentation of plasma membrane (arrowheads) are shown. (**B**) The graph reports the percentage of METH-induced cell death. (**C**) The graph reports the linear regression between the percentage of cell death and the percentage of altered mitochondria following increasing doses of METH (*p* = 0.04). Values are given as the mean percentage ± S.E.M. from N = 100 cells. * *p* ≤ 0.05 compared with controls; # *p* ≤ 0.05 compared with controls and METH 10 μM. Scale bars = 700 nm. N = nucleus.

Detection of METH-induced cell death by TEM provides a confirmation of data obtained at H&E staining. In fact, the dose response obtained by electron microscopy provides data that overlap with H&E light microscopy, where the dose of METH 10 μM produces slight changes that reach up to roughly 70% of cell loss at METH 1000 μM (representative Figure 5A and graph of Figure 5B), compared with roughly 20% of spared cells detected by H&E (Figure 1B). As shown in the representative pictures of Figure 5A, cell death is evidenced (where apoptosis and necrosis are concomitant). When the amount of cell death is compared with the occurrence of mitochondrial alterations a significant correlation (*p* = 0.04) is measured (Figure 5C). This correlation is carried out considering all mitochondrial alterations as detailed in the next paragraph.

### 2.4. Dose-Dependent Effects of METH on Mitochondral Alterations Detected at TEM

**Figure 6:**
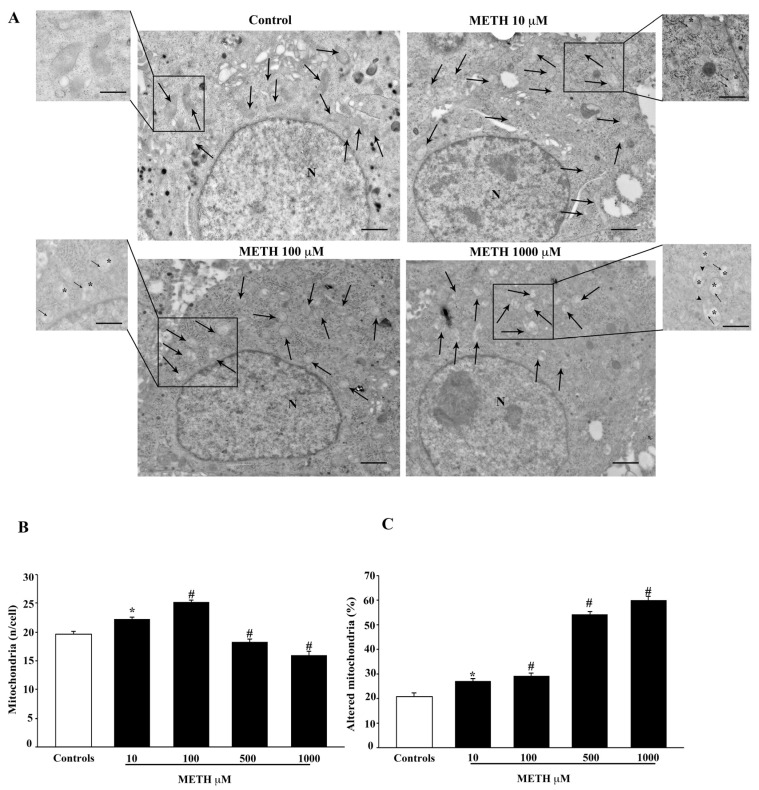
METH produces dose-dependent mitochondrial alterations. (**A**) Representative TEM micrographs showing mitochondria (arrows) in control and after increasing doses of METH. Inserts at high magnification show details of mitochondrial ultrastructure for each experimental group, such as matrix dilution (*), broken crests (arrows) and ruptures of membranes (arrowheads). (**B**) The graph reports the total number of mitochondria after increasing doses of METH (from 10 μM up to 1000 μM). (**C**) The graph reports the dose-dependent increase in altered mitochondria following METH. Values are given either as the mean ± S.E.M (**B**) or as the mean percentage ± S.E.M. (**C**) from N = 50 cells per group. * *p* ≤ 0.05 compared with controls; # *p* ≤ 0.05 compared with controls and METH 10 μM. Scale bars = 500 nm; 280 nm (inserts).

The number of total and altered mitochondria, counted by TEM indicates that METH produces dose-dependent effects, as shown in the representative pictures of Figure 6A. The effects of METH on the number of total mitochondria counted by TEM is reported in the graph of Figure 6B, which provides data quite similar to those obtained by light microscopy following MTR-G staining (graph of Figure 3B). In fact, both light and electron microscopy indicate that the highest number of mitochondria is counted for the dose of METH 100 μM. Similarly, the dose-dependent increase in the amount of METH-induced altered mitochondria counted by TEM (graph of Figure 6C) is consistent with the dose-dependent decrease of healthy mitochondria, which is reported following MTR-R staining (graph of Figure 4B). In fact, the number of healthy mitochondria stained with MTR-R falls significantly at doses of METH above 100 μM, which corresponds to a significant rise in altered mitochondria counted by TEM ultrastructural morphometry (graph of Figure 6C).

**Figure 7:**
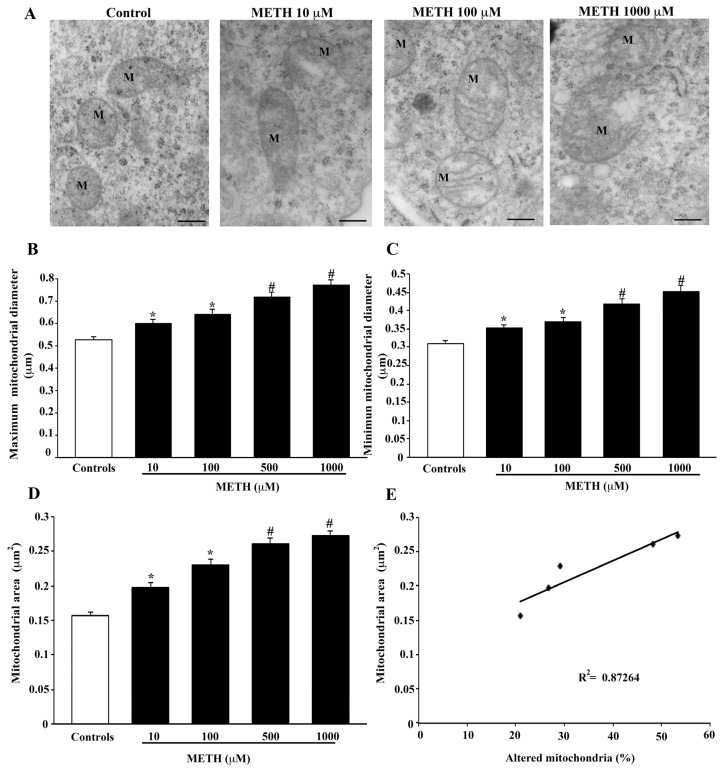
METH modifies mitochondrial size. (**A**) Representative TEM micrographs showing dose-dependent mitochondrial (M) changes induced by METH. Graphs report the maximum (**B**), and the minimum (**C**) mitochondrial diameter and the mitochondrial area (**D**). The graph reports the linear regression (**E**) between the mitochondrial area and the percentage of altered mitochondria for various doses of METH (*p* = 0.02). Values are given as the mean ± S.E.M from N = 150 mitochondria per group. * *p* ≤ 0.05 compared with controls; # *p* ≤ 0.05 compared with controls and METH 10 μM and 100 μM. Scale bars = 160 nm.

In order to express the number of altered mitochondria, specific features were analyzed, consisting of matrix dilution, broken crests and rupture of mitochondrial membranes. These alterations, when occurring either alone or in combination, were considered per se as a marker to define altered mitochondria. The mitochondrial area was not included among alterations selected for this specific analysis. This choice was based on the bias potentially occurring when a divergency between the cutting plane of the microtome and spatial arrangement of organelles in the slice takes place. Therefore, mitochondrial area was considered independently of mitochondrial alterations. The occurrence of each mitochondrial alteration does correlate with an increase in the mitochondrial area. In fact, as shown in representative Figure 7A, mitochondrial diameters and area all increased dose dependently following METH administration (representative Figure 7A). The count of maximum mitochondrial diameter (graph of Figure 7B) as well as the count of minimum mitochondrial diameter (graph of Figure 7C) all increased dose dependently following METH. As expected, such a dose dependency was enhanced by the measurement of mitochondrial area (graph of Figure 7D). When plotting the number of mitochondrial alterations vs. the mitochondrial area a significant correlation was drawn (graph of Figure 7E, *p* = 0.02).

**Figure 8:**
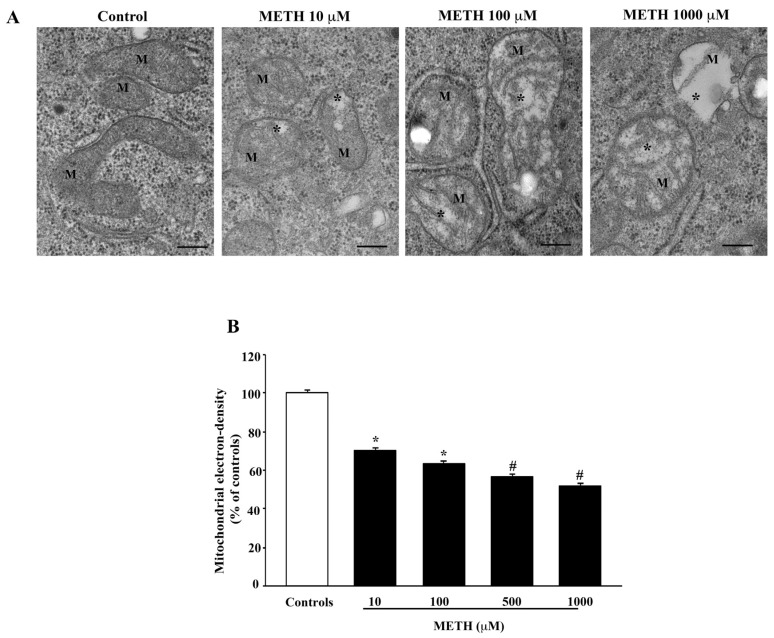
METH decreases matrix electron-density. (**A**) Representative TEM micrographs showing a dose-dependent decrease in electron density of mitochondrial matrix. In control, mitochondria possess a marked electron dense matrix, whereas after increasing doses of METH (from 10 μM up to 1000 μM) the mitochondria possess a diluted matrix that appears less electron dense compared with control (*). M = mitochondria. (**B**) Graph reports values showing matrix dilution as a weighted measurement (percentage of matrix electron density from controls). Values are given as the percentage mean ± S.E.M from N = 150 mitochondria per group. * *p* ≤ 0.05 compared with controls; # *p* ≤ 0.05 compared with controls and METH 10 μM and 100 μM. Scale bars = 160 nm.

**Figure 9:**
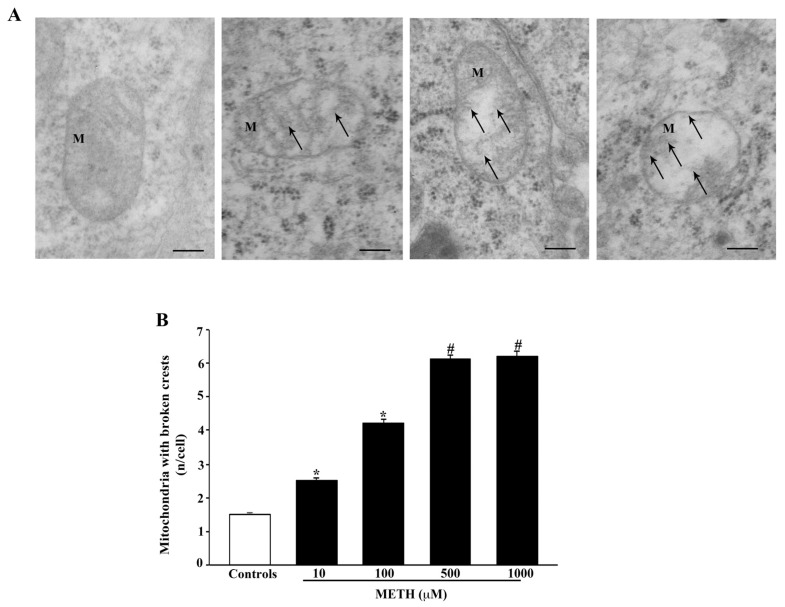
METH breaks mitochondrial crests. (**A**) Representative TEM micrographs showing that METH dose-dependently increases mitochondria (M) with broken crests (arrow). (**B**) The graph reports mitochondria with broken crests. Values are given as the mean ± S.E.M from N = 50 cells per group. * *p* ≤ 0.05 compared with controls; # *p* ≤ 0.05 compared with controls and METH 10 μM and 100 μM. Arrows point to broken crests. Scale bars = 160 nm.

**Figure 10:**
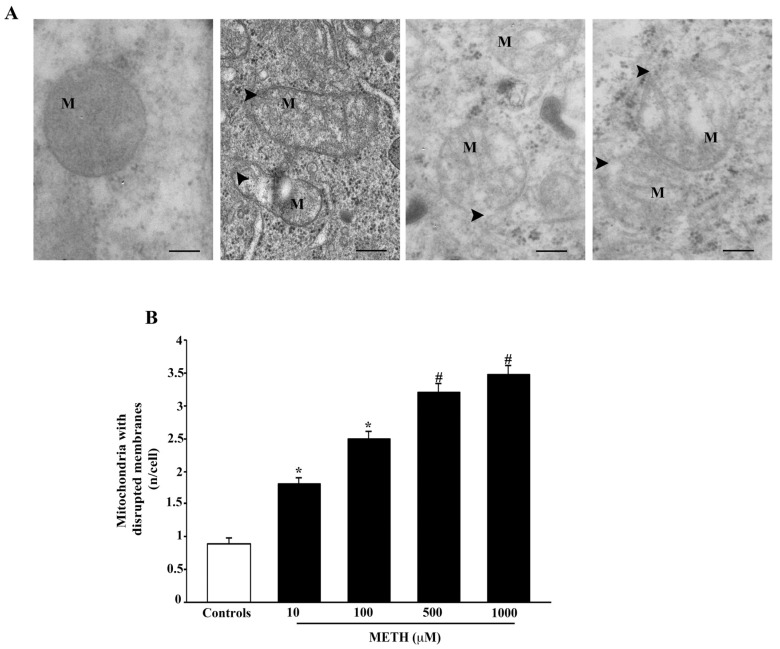
METH disrupts mitochondrial membranes. (**A**) Representative TEM micrographs showing that METH dose-dependently increases the ruptures of inner and outer membranes (arrowhead) of mitochondria (M). (**B**) The graph reports mitochondria with ruptured membranes. Values are given as the mean ± S.E.M from N = 50 cells per group. * *p* ≤ 0.05 compared with controls; # *p* ≤ 0.05 compared with controls and METH 10 μM and 100 μM. Scale bars = 160 nm.

Mitochondrial alterations were analyzed considering the following features: (i) loss of electron density in the mitochondrial matrix; (ii) number of mitochondria with broken crests; (iii) number of mitochondria with rupture of inner and/or outer membrane. It is remarkable that a consistency among all these measurements is measured, which correlates with the dose of METH (compare Figure 8 with Figure 9 and Figure 10). When considering the electron density of the mitochondrial matrix, this is reduced dose dependently by METH, as shown in representative Figure 8A and calculated in the graph of Figure 8B. Such a decrease occurs even for the lowest dose of METH, 10 μM, and progresses up to 500 μM and 1000 μM.

Broken crests within mitochondria are shown in the representative pictures of Figure 9A. The amount of these mitochondrial alterations was calculated in the graph of Figure 9B.

Even in this case the doses of METH 500 μM and METH 1000 μM produce the maximal effects, which is already significant at the lowest dose of METH 10 μM. The rupture of the internal and external mitochondrial membrane is evidenced in the representative pictures of Figure 10A, whereas the dose-response effects of METH on this alteration are reported in the graph of Figure 10B. The dose-response curve is similar to that described for the occurrence of broken crests.

Thus, each elementary alteration of mitochondrial structure is significantly evident for the lowest dose of METH, which does not produce significant cell loss but does produce significant cell degeneration. These data lend substance to these alterations as highly predictive markers of mitochondrial integrity and METH-induced degeneration rather than cell death.

**Figure 11:**
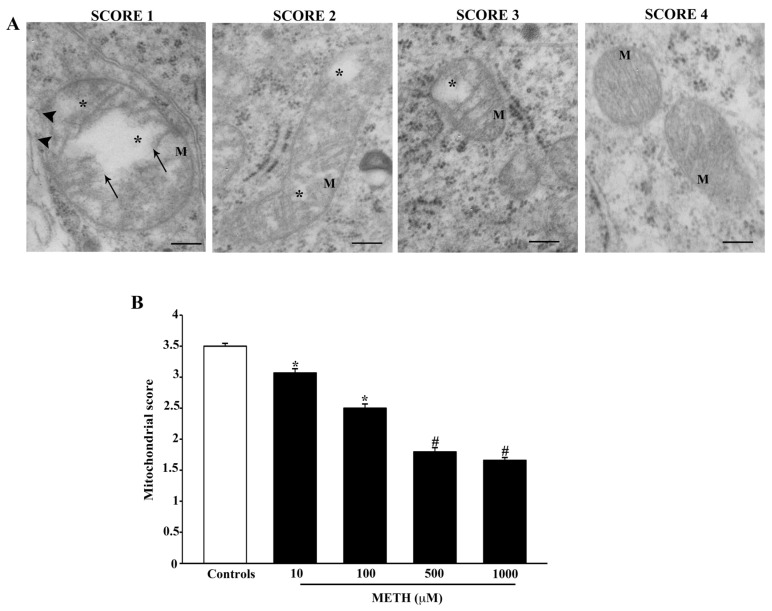
METH decreases the mitochondrial integrity score. (**A**) Representative TEM micrographs showing that METH dose-dependently decreases the integrity of those features used to assess the mitochondrial integrity score. Arrows point to broken crests; arrowheads point to ruptured membranes; * indicates matrix dilution. (**B**) The graph reports the non-parametric mitochondrial integrity score from N = 150 mitochondria per group. Assuming the score as the sum of four non-parametric values, the parametric mean of these values is provided along with its standard error. In this way, the means are compared inferentially. * *p* ≤ 0.05 compared with controls; # *p* ≤ 0.05 compared with controls and METH 10 μM and 100 μM. Scale bars = 160 nm.

Therefore, as part of the results of the present study we provide a novel assessment of mitochondrial damage, which combines these elementary mitochondrial alterations to provide an experimental tool to score mitochondrial integrity. The present assessment extends previous studies [38] and it is defined as follows: Stage 1 is characterized by the concomitant occurrence of broken crests, with ruptured membranes and widespread matrix dilution. Stage 2 is characterized by the concomitance of broken crests and spots of matrix dilution. Stage 3 features spots of matrix dilution only. Stage 4 refers to intact mitochondria. This mitochondrial score decreases dose dependently with increasing METH doses (Figure 11). The score is already diminished at the lowest dose of METH, 10 μM (Figure 11B).

### 2.5. Effects of METH In Situ on Ultrastructural Stoichiometry of Proteins Affecting Mitochondrial Dynamics

**Figure 12:**
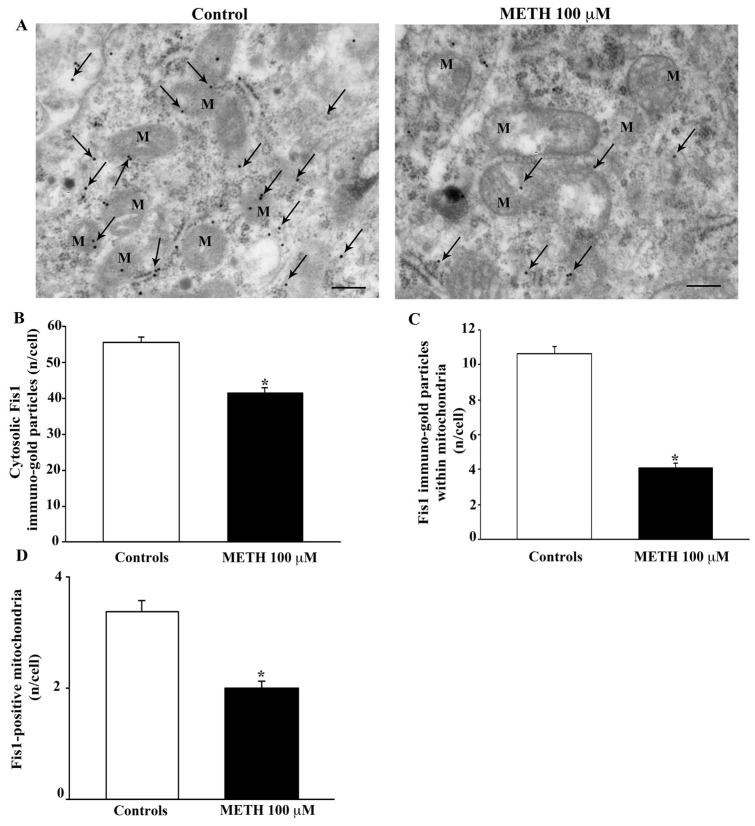
METH decreases the fission protein Fis1. (**A**) Representative TEM micrographs showing Fis1 particles from control and following METH 100 μM. Arrows point to Fis1 immunogold particles within cytosol and mitochondria (M). Graph (**B**) reports the number of Fis1 immunogold particles within the cytosol. Graph (**C**) reports the number of Fis1 immunogold particles within mitochondria. Graph (**D**) indicates the number of Fis1-positive mitochondria. Values are given as the mean ± S.E.M from N = 50 cells per group. * *p* ≤ 0.05 compared with controls. Scale bars = 170 μm.

To provide a further insight on changes in protein levels, which sustain mitochondrial dynamics, in situ ultrastructural morphometry was carried out for a few proteins involved in mitochondrial fission and mitophagy. As shown in the representative pictures of Figure 12A the amount of the protein Fis1, which is mostly involved in mitochondrial fission and partly in mitophagy, is decreased following the dose of METH 100 μM. The amount of Fis1 is significantly decreased in the cytosol and mostly in situ, within mitochondria, where the protein falls to roughly 50% of control as calculated in the graphs of Figure 12B–D. This is confirmed by Western blotting (Supplementary Figure S1) and immunofluorescence (Supplementary Figure S2).

**Figure 13:**
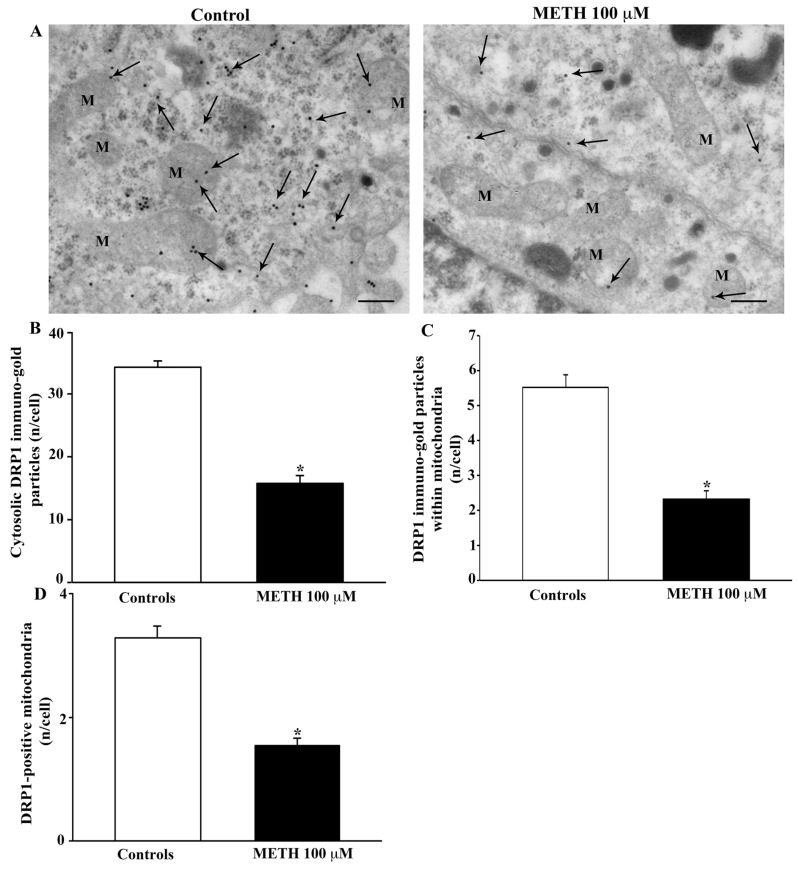
METH decreases the fission protein DRP1. (**A**) Representative TEM micrographs showing DRP1 particles from control and following METH 100 μM. Arrows point to DRP1 immunogold particles within cytosol and mitochondria (M). Graph (**B**) reports the number of DRP1 immunogold particles within the cytosol. Graph (**C**) reports the number of DRP1 immunogold particles within mitochondria. Graph (**D**) indicates the number of DRP1-positive mitochondria. Values are given as the mean ± S.E.M from N = 50 cells per group. * *p* ≤ 0.05 compared with controls. Scale bars = 170 μm.

When immunogold detection was directed against DRP1, this protein, which is equally involved in mitochondrial fission and mitophagy, is reduced by METH as shown in the representative pictures of Figure 13A and is counted in the graphs of Figure 13B–D. The amount of such a decrease is similar within the cytosol and in situ, at mitochondrial level.

**Figure 14:**
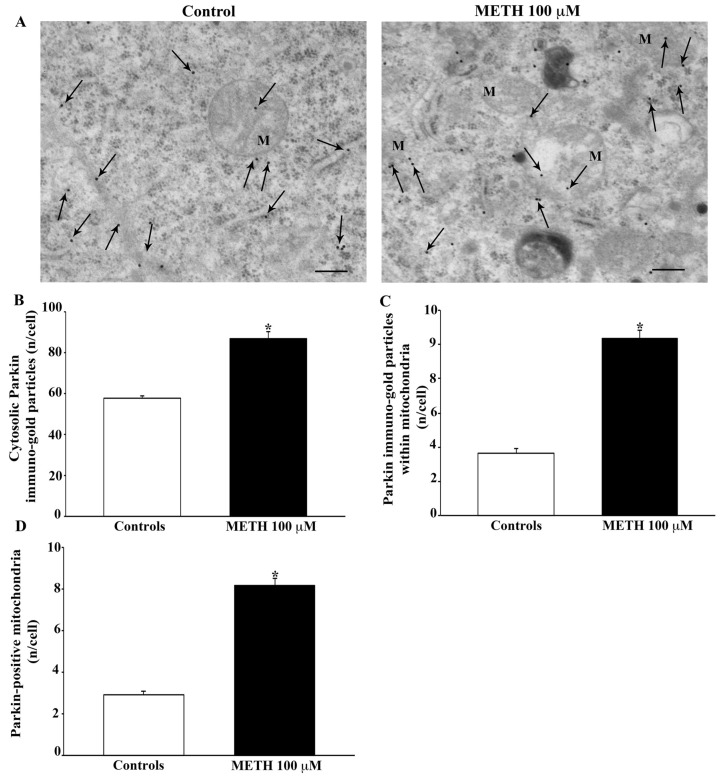
METH increases the protein, Parkin. (**A**) Representative TEM micrographs showing Parkin particles from control and following METH 100 μM. Arrows point to Parkin immunogold particles within cytosol and mitochondria (M). Graph (**B**) reports the number of Parkin immunogold particles within the cytosol. Graph (**C**) reports the number of Parkin immunogold particles within mitochondria. Graph (**D**) indicates the number of Parkin-positive mitochondria. Values are given as the mean ± S.E.M from N = 50 cells per group. * *p* ≤ 0.05 compared with controls. Scale bars = 170 μm.

In Figure 14A, the enzyme E3-ubiquitin ligase, Parkin, is shown in representative pictures. The dose of METH 100 μM increases Parkin both in the cytosol and in situ, within mitochondria (representative pictures of Figure 14A). The increase is much more evident in situ at the mitochondrial level than in the cytosol as counted in the graphs of Figure 14B–D. Western blotting confirms the increase in Parkin (Supplementary Figure S3).

**Figure 15:**
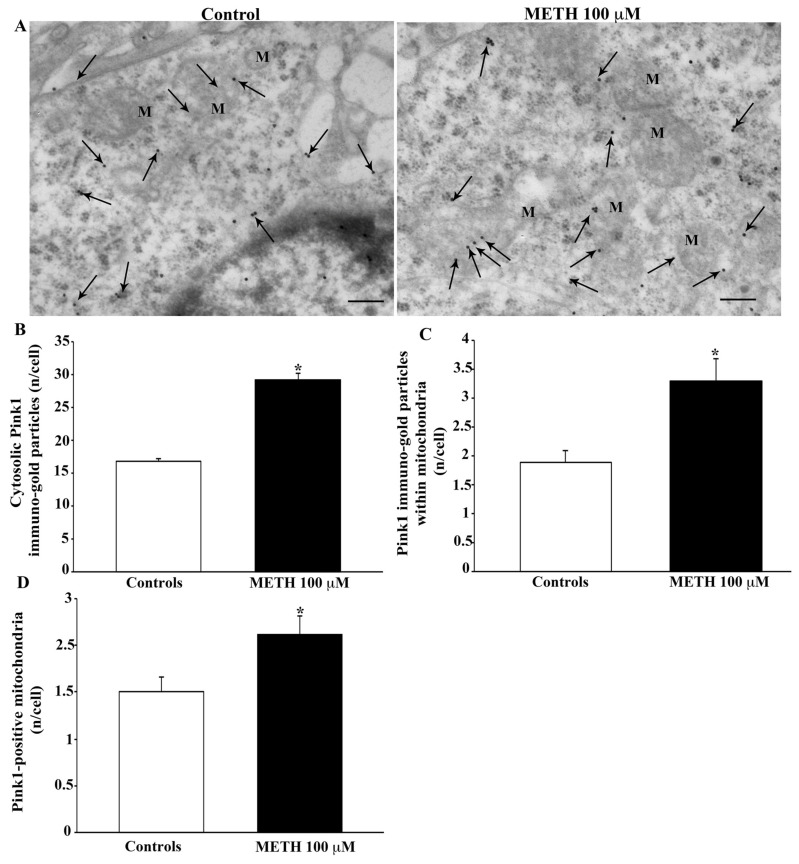
METH increases the protein, Pink1. (**A**) Representative TEM micrographs showing Pink1 particles from control and following METH 100 μM. Arrows point to Pink1 immunogold particles within cytosol and mitochondria (M). Graph (**B**) reports the number of Pink1 immunogold particles within the cytosol. Graph (**C**) reports the number of Pink1 immunogold particles within mitochondria. Graph (**D**) indicates the number of Pink1-positive mitochondria. Values are given as the mean ± S.E.M from N = 50 cells per group. * *p* ≤ 0.05 compared with controls. Scale bars = 170 μm.

The presence of Pink1, which is very scattered in the cytosol, was increased following administration of METH 100 μM, as shown in the representative pictures of Figure 15A. This METH-induced increase in Pink1 was more pronounced in situ, within mitochondria, compared with cytosol, as counted in the graphs of Figure 15B–D. This is also confirmed by Western blotting (Supplementary Figure S4).

This is confirmed by immunofluorescence. In fact, METH increases both Pink1 and Parkin immunofluorescence dose dependently (Supplementary Figure S5). Additionally, representative pictures show increased merging of Pink1 and Parkin immunofluorescence (Supplementary Figure S5A). Immunogold by TEM confirms co-localization of Pink1 and Parkin specifically within mitochondria (Supplementary Figure S6).

## 3. Discussion

The present study indicates that METH markedly alters mitochondria. This is evidenced at first by light microscopy, which indicates that METH dose-dependently reduces the number of healthy mitochondria despite increasing the number of total mitochondria when cells keep their structural integrity. The increase in total mitochondria was measured by MTR-G histofluorescence. Since MTR-G staining does not distinguish between altered and healthy mitochondria, an increase in MTR-G histofluorescence needs to be interpreted considering the decrease in healthy mitochondria detected by MTR-R. This leads us to consider such an increase as due to a higher number of altered organelles. In addition, since MTR-G histofluorescence does not allow detection of a single organelle, it is likely that an increased mitochondrial area, which occurs following METH, may lead MTR-G staining to overestimate the mitochondrial number. In fact, when mitochondria are directly identified by TEM, altered mitochondria possess a significant enlargement. Thus, the occurrence of an increase in total mitochondria, as detected using MTR-G, needs to be corrected considering that, under the effects of METH, each mitochondrion measures an increased area. Since MTR-G does not allow counting of single mitochondria, TEM morphometry is useful to understand the real increase in the number of mitochondria. This explains why, semi-quantitative assessment of mitochondria, which is measured using MTR-G, leads to an excess of 200% of control, whereas the authentic number of mitochondria counted by TEM is in excess of 25% of control. Thus, under the effects of METH total mitochondria undergo a significant increase, whereas healthy mitochondria are significantly decreased. The increase in altered mitochondria documented by TEM is due to the occurrence of specific structural alterations, such as matrix dilution, broken crests, and rupture of both inner and outer membranes. These elementary alterations are already present at the lowest dose of METH, 10 μM, which fully correlates with the neurodegeneration detected by FJB histofluorescence. In fact, the occurrence of cell death is significant only starting from the dose of METH 100 μM. In turn, the occurrence of mitochondrial alterations is correlated with an increase in mitochondrial area, which reaches two-fold of controls. When considering that the number of mitochondria in METH-treated cells increases in excess of 25% of controls and the mean mitochondrial area measured following METH is twice the area of controls, the increase in mitochondrial fluorescence detected by MTR-G is much more plausible. In order to further discuss the significance of data obtained by MTR-G, one should also consider that when such a histofluorescence is carried out, the amount does not simply reflect well-defined mitochondria, which instead are counted by TEM. In fact, MTR-G is also affected by mitochondria within autophagy vacuoles or within the lysosomal compartment [39], which in turn are amplified by METH administration, which produces large stagnant autophagosomes where mitochondrial clearance is impaired [40]. Again, some MTR-G fluorescence may be produced by mitochondrial remnants and endoplasmic reticulum [41], which are also increased following METH.

Thus, the present study indicates that METH administration dose-dependently increases catecholamine cell death and mostly (starting at lower doses) cell degeneration (see also Supplementary Figure S7). These effects (mostly cell degeneration) are highly correlated with the occurrence of mitochondrial alterations, enlargement of mitochondrial area and the decrease in healthy mitochondria. In fact, occurrence of cell death is mostly severe, and it does not allow those slowly progressing cyto-pathological effects, which are evident during a degenerative phenomenon.

The dose of METH used in the present study ranges from 10 μM to 1000 μM. The doses were selected based on the biochemical effects induced in catecholamine cells. This comparison was carried out by Fornai et al., 2007 [35], who found that a higher dose of METH was needed in vitro to produce the same effects in vivo. In fact, these doses of METH lead to molarities within DA-containing brain areas that were lower than the molarity that is needed to produce comparable effects in vitro.

A number of studies have characterized the doses of METH in vitro that are comparable to the doses administered in vivo. In fact, toxicity for DA neurons occurs for cumulative doses of METH that are administered to rodents of about 25 mg/kg. Interestingly, as extensively addressed by some recent reviews, the doses of METH administered to rodents ranged cumulatively from 5 mg/kg up to 25 mg/kg, which corresponds to the wide range of the METH intake by humans [4,42]. These doses of METH lead to a molarity within DA-containing brain areas that are lower than the molarity that is needed to produce comparable effects in vitro. METH doses from 1 μM up to 2000 μM are commonly used in the literature to document at cellular level the molecular mechanisms of action of METH and its neurotoxicity [14,27,43,44,45,46,47]. These doses are similar both in undifferentiated and differentiated PC12 cells, SH-SY5Y cells or other types of cell cultures. The difference between in vitro and in vivo conditions are likely to be due to the intrinsic features of PC12 cells [35]. These include the following: (i) the presence of VMAT-1, which is less specific for the vesicular uptake of catecholamines when compared with its homolog, VMAT-2, expressed in the brain, and (ii) low levels of the DAT, thus reduced cytosolic reuptake of DA [35].

A number of studies demonstrate that METH alters mitochondria both through a direct and indirect effect. Most of these studies are recent and they focused on the impairment of mitochondrial activity [31,32,33,34], thus leaving unexplored the alterations in mitochondrial morphology induced by METH. In the present study, mitochondrial alterations were detailed by ultrastructural morphometry. This allowed substantiation of the fine morphological changes that occur following METH exposure.

The following mitochondrial alterations were detailed: (i) loss of electron density in the mitochondrial matrix; (ii) number of mitochondria with broken crests; (iii) number of mitochondria with rupture of inner and/or outer membrane. When counting these alterations, it is evident that all of them correlate with cell loss. However, these alterations begin to be present at the lowest dose of METH, 10 μM, which fully overlaps with the occurrence of METH-induced neurodegeneration. Again, we noticed that occurrence of one type of mitochondrial alteration prevails for more minor mitochondrial damage, whereas serious damage features all types of alterations. Therefore, we suggested a novel scoring system that enlarged the previous scoring suggested by Flameng et al. (1980) [38]. The revised score for mitochondrial integrity goes from severe to slight damage: Stage 1 is characterized by the concomitant occurrence of broken crests, with the rupture of outer and inner membranes and widespread matrix dilution. Stage 2 is characterized by the concomitance of broken crests and spots of matrix dilution. Stage 3 features spots of matrix dilution only. Stage 4 refers to intact mitochondria.

The occurrence of METH-induced mitochondrial damage is correlated with increased mitochondrial area. Thus, it is not surprising that matrix dilution occurring in spots or widespread within mitochondria progresses along with mitochondrial damage and enlargement. The increase in mitochondrial area is supposed to be symmetrical in shape since the increase in all mitochondrial diameters occurs to a similar extent. The persistence of altered giant mitochondria within degenerating METH-treated cells is supposed to depend on a defect in mitochondrial dynamics such as the impairment of mitochondrial fission or mitochondrial removal through effective mitophagy. In fact, when specific markers for these phenomena were quantified in METH-treated cells and in situ within mitochondria, data were consistent with such a hypothesis. In detail, the fission-related protein, Fis1, as well as the fission/mitophagy marker, DRP1, were decreased. Such a decrease, which is significant in the whole cytosol, is markedly significant in situ, within mitochondria. In fact, in the present study proteins were authentically measured using ultrastructural stoichiometry, where a single immunogold particle stains a single protein. Such an approach allows counting of the protein specifically within mitochondria and to compare whole cell protein variations with focal amounts counted within these organelles. The decrease in the fission proteins Fis1 and DRP1 is consistent with the effects of METH, which induces multiple damage to the mitochondrial respiratory chain [31,32,33]. In fact, the suppression of these proteins sustaining the fission machinery is supposed to accumulate dysfunctional mitochondria [48]. In fact, we measured an increase in the classic sensor for mitochondrial damage, Pink1, following METH administration. Such an increase is more evident within mitochondria than cytosol. Since mitochondrial expression of Pink1 is related to the mitochondrial recruitment of Parkin to deliver altered organelles to lysosome clearance [49,50], we tested the amount of Parkin in the same experimental conditions. In fact, Pink1 and Parkin are proteins that are key regulators of mitochondrial removal. Their concomitant binding to mitochondria occurs when mitochondria are altered. In fact, in the presence of mitochondrial damage, Pink1 binds to mitochondria to recruit Parkin. Therefore, co-localization of Pink1 and Parkin on the mitochondria occurs specifically in the presence of mitochondrial alterations [51,52,53]. Moreover, Pink1/Parkin are involved in modulating the balance between mitochondrial fission and fusion [52,54,55].

As expected, the amount of Parkin within mitochondria is increased following METH administration and it surpasses the increase in Parkin measured within cytosol. This is consistent with previous data showing that Pink1 overexpression leads to Parkin accumulation within mitochondria, whereas silencing Pink1 erases Parkin from mitochondria [49]. The persistency of altered mitochondria in METH-treated cells is dose dependent and it is likely to be related to a defect placed downstream in mitochondrial removal. In fact, Burman et al. (2017) [56] found that a loss of DRP1 is concomitant to, and induces, the recruitment of Parkin to mitochondria.

Only a few studies have focused on mitochondrial alterations during METH toxicity [46,57]. When dealing with fine alterations of mitochondrial ultrastructure only a few reports are available [58]. Previously we approached such an issue by analyzing the effects of METH on mitochondrial ultrastructure in the presence of different variants of the gene expressing *PINK1*, which leads to genetic parkinsonism [49]. In this study, we hypothesized that Pink1 placed at the mitochondrial level may act as a sensor of mitochondrial damage to recruit mitochondrial removal via interaction with the protein, Parkin, which is largely used to analyze the mitochondrial status. This study indicates for the first time that METH increases Parkin at the mitochondrial level. Thus, in conditions of strong oxidative stress that produce a mitochondrial failure, it is likely that Pink1 recruits Parkin at the surface of dysfunctional mitochondria in an attempt to activate the mitophagy cascade. In fact, we documented that alterations in Pink1 fail to recruit Parkin and produce an altered mitochondrial homeostasis. In fact, when Pink1 is removed from the cell, accumulation of mitochondria owing to severe damage occurs, along with apoptotic cells [49].

The present data provide a novel scenario for the intracellular alterations occurring following METH, which indicates that mitochondrial damage is involved in METH-induced alterations of neural activity. The damage that is induced in the present experimental setting is likely to occur in vivo in the addicted brain and during specific neurodegenerative conditions that are mimicked by METH [4]. This is the case of degenerative cognitive decline and parkinsonian movement disorders. In all these conditions severe mitochondrial alterations can be documented and they are considered as a culprit for the onset and progression of the disease. Thus, targeting mitochondria to prevent their damage and improving the removal of altered mitochondria through compounds that stimulate mitophagy could be a promising strategy to treat these disorders.

## 4. Materials and Methods

### 4.1. Cell Cultures

Pheochromocytoma PC12 cells were purchased from IRCCS San Martino Institute (Genova, Italy). Cells were grown in RPMI 1640 medium (Sigma-Aldrich, St. Louis, MO, USA), supplemented with horse serum (HS, Sigma-Aldrich), fetal bovine serum (FBS, Sigma-Aldrich), and antibiotics, in a wet atmosphere with 5% CO_2_ at 37 °C. Experiments were carried in the log-phase of growth, which corresponds to 70% confluence [59,60]. Cells were seeded and incubated at 37 °C in 5% CO_2_ for 24 h before treatment.

PC12 cells were treated for 72 h (this time interval was selected based on previous studies, refs. [40,44,61]) with culture medium with or without increasing doses of METH, ranging from 50 μM up to 1000 μM. In detail, the stock solution of METH (kindly gifted by Forensic Medicine, University of Pisa), 10 mM, was prepared by dissolving 2.3 mg of METH in 1 mL of culture medium. The treatment solutions were obtained by diluting aliquots of the stock solution in the culture medium. Cell cultures were exposed continuously for 72 h to METH. Control cultures were administered the same volumes of the culture medium for 72 h without being washed. At the end of this time interval various dishes of PC12 cells were processed according to the various experimental procedures.

### 4.2. Hematoxylin and Eosin (H&E) Histochemistry

For H&E staining, 5 × 10^4^ PC12 cells were seeded on poly-lysine coverslips and placed in 24-well plates in a final volume of 1 mL/well.

After fixation in a 4% paraformaldehyde phosphate buffered solution (PBS) for 15 min, PC12 cells were washed in PBS and immersed in the hematoxylin solution (Sigma-Aldrich) for some minutes. After stopping the hematoxylin staining through repeated washing, cells were plunged within the eosin solution (Sigma-Aldrich), washed out again to remove the excess of dye and then dehydrated in increasing alcohol solutions. Finally, cells were clarified in xylene, covered with DPX mounting medium (Sigma-Aldrich) and observed under a Nikon Eclipse 80i light microscope (Nikon, Tokyo, Japan).

Cell counting was performed by light microscopy at 20× magnification; the number of stained cells detectable after each specific treatment was counted and expressed as the mean percentage ± SEM of the control group (which corresponds to 100%). Data refer to three independent experiments.

### 4.3. Fluoro-Jade B (FJB) Histofluorescence

For Fluoro-Jade B (FJB) staining [37] 5 × 10^4^ PC12 cells were seeded on poly-lysine coverslips and placed in 24-well plates in a final volume of 1 mL/well.

After washing in PBS, cells were fixed with a solution of paraformaldehyde 4% for 5 min and incubated with 0.06% potassium permanganate for 10 min at room temperature. After washing in distilled water, cells were incubated for 20 min in 0.0004% FJB (Merck Millipore, Billerica, MA, USA) solution (consisting of 0.01% FJB in acetic acid) and cover slipped with mounting medium. FJB-positive cells were analyzed using a Nikon Eclipse 80i light microscope (Nikon, Tokyo, Japan), equipped with a florescence lamp and a digital camera connected to the NIS Elements software for image analysis (Nikon, Tokyo, Japan), where the count of FJB-positive cells was carried out at 20× magnification. Values were expressed as the mean number ± SEM for each experimental group. The intensity of the fluorescent signaling was measured under florescence microscopy using the software, Image J (NIH, USA, Version 1.8.0_172), and values are expressed as the mean percentage ± S.E.M. of optical density (assuming controls as 100%) from N = 90 cells/group. All data refer to three independent experiments.

### 4.4. Trypan Blue (TB) Staining

TB staining was carried out in PC12 cells, which were seeded 24 h before treatment at a density of 10^4^ cells/well and placed within 24-well plates in 1 mL of culture medium. After treatment, cells were collected, centrifuged at 800× *g* for 5 min and the cell pellet was suspended in 0.5 mL of culture medium. Twenty-five μL of cell suspension was incubated for 10 min in a solution containing 1% TB (62.5 μL) and PBS (37.5 μL). A 10 μL aliquot of this solution was placed in a Bürker chamber and analyzed under an Olympus CKX 41 inverted microscope (Olympus Corporation, Tokyo, Japan), where viable and nonviable cells were counted. Values were expressed as the mean percentage ± S.E.M. of TB-positive cells out of the total cells. Data represent the means of three chamber counts, from three independent experiments.

### 4.5. Mitochondrial Labeling

To stain mitochondria in living cells, MitoTracker Red or MitoTracker Green (MTR-G), which stain healthy or total (both healthy and unhealthy) mitochondria, respectively, were used [62,63,64]. Briefly, 5 × 10^4^ PC12 cells were grown in 24-well plates containing 1 mL/well of culture medium. Cells were incubated in a solution of MTR-R (Thermo-Fisher Scientific, Waltham, MA, USA) or MTR-G (Thermo-Fisher Scientific) at 500 nM in a serum-free culture medium for 45 min, at 37 °C and 5% CO_2_. Then, staining solutions were removed and fresh pre-warmed medium was added. Cells were immediately analyzed by fluorescence microscopy (Nikon). The optical density was measured under a fluorescence microscope using Image J software (NIH, USA, Version 1.8.0_172). Values are given as the mean percentage ± S.E.M. of the optical density (assuming controls as 100%) measured in N = 90 cell/group. All data refer to three independent experiments.

### 4.6. Immunocytochemistry by Light Microscopy

For light microscopy, 5 × 10^4^ cells were placed on poly-lysine slides in 24-well plates with 1 mL/well of culture medium. Cells were washed in PBS and fixed with 4% paraformaldehyde in PBS for 15 min, followed by 0.1% TritonX-100 for 15 min in PBS and 10% normal goat serum in PBS for 1 h at room temperature. Cells were then incubated overnight at 4 °C in 1% normal goat serum in PBS containing the primary antibodies (diluted 1:50) as follows: (i) anti-Pink1 (Abcam, Cambridge, UK) + anti-Parkin (Millipore, Burlington, MA, 808 USA) antibodies; (ii) anti-mitochondrial fission 1 protein (Fis1, GeneTex, Irvine, CA, USA) antibodies. After washing in PBS, cells were incubated at for 1 h with a 1:200 dilution of appropriate fluorophore-conjugated secondary antibodies, namely anti-rabbit Alexa488 and anti-mouse Alexa594 (Life Technologies, Carlsbad, CA, USA). The fluorescent dye, DAPI (Sigma-Aldrich), was used to stain nuclei. Then, cells were washed in PBS and gently transferred to a coverslip to be finally mounted with the mounting medium Fluorosheild (Sigma-Aldrich). Slides were observed using a Nikon Eclipse 80i light microscope, which was equipped with a fluorescent lamp and a digital camera connected to the NIS Elements Software for image analysis (Nikon, Tokyo, Japan). Control sections were incubated with secondary antibodies only. The optical density was measured using Image J software (1.8.0_172, NIH, Bethesda, MD, USA). Values are given as the mean percentage ± S.E.M. from N = 90 cells/group.

### 4.7. Transmission Electron Microscopy (TEM)

For transmission electron microscopy (TEM), 1 × 10^6^ cells were seeded in culture dishes in a final volume of 5 mL. After 5 min centrifugation at 1000× *g*, cell pellets were rinsed in PBS and fixed for 90 min at 4 °C in a solution of 2.0% paraformaldehyde and 0.1% glutaraldehyde in 0.1 M PBS (pH 7.4). After washing in PBS (0.1 M), samples were post-fixed in 1% osmium tetroxide (OsO_4_) for 1 h, at 4 °C, then specimens were dehydrated in ethanol solutions (30%, 50%, 70%, 90% and 95%, and 100%) and they were embedded in epoxy resin.

Ultra-thin slices (90 nm thick) were cut by ultra-microtome (Leica Microsystems, Wetzlar, Germany), and they were counterstained with uranyl acetate and lead citrate and dissolved in distilled water to be examined using a JEOL JEM SX100 transmission electron microscope (JEOL, Tokyo, Japan).

### 4.8. Ultrastructural Morphometry of Mitochondria

Grids of ultrathin sections (90 nm thick) were magnified at 6000× to count both total and altered mitochondria in each cell. A number of grids was counted to harvest at least 50 cells per group. Starting from a corner, the whole embedded pellet within that grid was scanned in parallel sweeps. Mitochondria were easily identified by TEM, consisting of a typical double-membrane limiting an inter-membrane (“inter-mitochondrial”) space and an area internal to the inner membrane, where the matrix is interrupted by crests, in a sort of labyrinth. Although the morphology of mitochondria is standardized, many variations may be noticed at high magnification within specific cell cytopathologies [64,65].

The electron density in the mitochondrial matrix, mitochondrial diameters and area were measured along with broken crests and disrupted membranes with ImageJ software (NIH, USA, Version 1.8.0_172). Electron density was measured at 6000×. The mitochondrial electron density was normalized to the electron density of the cytosol. Measurement of the mitochondrial area was carried out using the tool “freehand selection” of Image J and reported as μm^2^.

### 4.9. Immunoelectron Microscopy

Post-embedding immunoelectron microscopy was validated in previous studies [40,49,66,67] by using OsO_4_ and epoxy resin to preserve ultrastructural morphometry. In fact, a combination of aldehydes, OsO_4_, and epoxy resin allows minimal epitope covering while preserving sub-cellular architecture [49,66,67,68]. Since OsO_4_ binds to lipid membranes, they are well stained compared with surrounding tissue. This allows detection of organelle contours and magnifies the subcellular trim. The use of epoxy compared with acrylic resin better preserves cell architecture.

Post-embedding immunoelectron microscopy was carried with gold-conjugated secondary antibodies allowing stoichiometric detection and placement of proteins within specific structures [69]. Ultrathin sections were collected on nickel grids and processed for protein detection. The oxidizing agent, sodium metaperiodate (NaIO_4_), was used to remove OsO_4_ as much as needed to unmask antigens [70]. The sodium metaperiodate attacks the hydrophobic alkane sidechains of epoxy resin thus making sections more hydrophilic and allowing a close contact between immunogold-conjugated antibodies and antigens [66,67]. Nickel grids were incubated with aqueous saturated NaIO_4_ solution for 30 min, at 21 °C. The primary antibodies used are the following: (i) Parkin (Millipore), (ii) Pink1 (Abcam), (iii) Fis1 (GeneTex, Irvine, CA, USA) and (iv) DRP1 (Abcam). All primary antibodies were used at a dilution of 1:20.

After washing in PBS, grids were incubated on drops of blocking buffer (10% goat serum and 0.2% saponin in PBS) for 20 min, at 21 °C, and then with a single primary antibody. Incubations were carried out on drops of ice-cold solution (PBS containing 1% goat serum and 0.2% saponin) in a humidified chamber overnight at 4 °C. After washing in cold PBS, slices were incubated on drops of blocking buffer (1% goat serum and 0.2% saponin in PBS) containing gold-conjugated secondary antibodies (10 nm or 20 nm gold particles, BB International, Treviso, Italy) diluted 1:20, for 1 h at 21 °C. After rinsing in PBS, grids were incubated with 1% glutaraldehyde for 3 min, and they were washed in distilled water to remove salt traces and prevent uranyl-acetate precipitation. Grids were counterstained with a saturated solution in distilled water of uranyl acetate and lead citrate. To control for method accuracy some slices were incubated with secondary antibody only.

### 4.10. Western Blotting

A total of 1 × 10^6^ PC12 cells were seeded in culture dishes in a final volume of 5 mL. After 5 min centrifugation at 1000× *g*, the cell pellet was placed in an Eppendorf tube containing 20 μL of ice-cold lysis buffer with phosphatase and protease inhibitors, to be homogenized. An aliquot of the homogenate was used for Bradford protein assay. Proteins (20 μg) were separated on SDS-polyacrylamide gels (Mini Protean TGX precast gel 4–20% gradient, BioRad Laboratories) on a Trans-blot Turbo Transfer System Pack (for mixed molecular weight; 1.3 A-25 V-10 min). Membranes were blocked for 2 h in Tween-20 Tris-buffered saline (TTBS) (100 mM Tris-HCl, 0.9% NaCl, 1% Tween 20, pH 7.4) containing 5% non-fat dry milk (BioRad Laboratories). The following primary antibodies were used: (i) anti-Tyrosine Hydroxylase (TH, 1:1000 Sigma-Aldrich), (ii) anti-Pink1 (1:1000, Abcam), (iii) anti-Parkin (1:1000, Termo Fisher Scientific, Monza (MB), Italy), (iv) anti-Fis1 (1:1000, GeneTex). Rabbit anti-β-actin (1:50,000; Sigma-Aldrich) was used as an internal standard for semi-quantitative protein measurement.

Membranes were incubated overnight at 4 °C with the solutions containing primary antibodies diluted in TTBS containing 2.5% non-fat dry milk. After washing in TTBS they were incubated for 1 h with peroxidase-labeled secondary antibodies (anti-rabbit/anti-mouse, 1:3000; Calbiochem, Milan, Italy). Bands were visualized with enhanced chemiluminescence reagents (GE Healthcare, Milan, Italy) and image analysis was carried out by the ChemiDoc System (Bio-Rad Laboratories). Optical density was normalized for relative β-actin using Image J software (NIH, USA, Version 1.8.0_172).

### 4.11. Statistical Analysis

For cell viability experiments, the number of H&E-stained cells was expressed as the mean percentage ± SEM of cells counted from three independent experiments (assuming controls as 100%).

TB-positive cells were expressed as the mean percentage ± S.E.M. of TB-positive cells out of the total cells, counted from three independent experiments.

The number of FJB-positive cells were expressed as the mean ± S.E.M. of FJB-positive cells counted from three independent experiments.

For FJB, MTR-R and MTR-G as well as Pink1, Parkin, and Fis1 immunofluorescence, the optical density of each sample was calculated. Values were expressed as the mean percentage ± S.E.M. of the fluorescent densitometry compared with control, which was measured in N = 90 cells/group from three independent experiments.

The optical density of immunoblotting for Fis1, Pink1 and TH was given as mean ± S.E.M. from 4 ≤ N ≤ 5 samples per experimental group.

Cell death measured by TEM was expressed as the mean percentage ± S.E.M. counted in N = 100 cells per group.

For ultrastructural morphometry, the number of total and altered mitochondria per cell were expressed as the mean and the mean percentage ± S.E.M. per cell, respectively (N = 50 cells per group).

Mitochondrial diameters and area, along with mitochondrial electron density were expressed as the mean ± S.E.M. from N = 150 mitochondria per group. Mitochondrial diameters and area were expressed as absolute number, mitochondrial electron density was weighted compared with the cytosol.

Broken crests and disrupted membranes of mitochondria were expressed as the mean ± S.E.M. from N = 50 cells per group.

Finally, the number of immunogold particles related to Fis1, DRP1, Pink1, Parkin and Pink1 + Parkin proteins were expressed as the mean ± S.E.M. from N = 50 cells per group.

Comparisons among different groups were carried out by one-way analysis of variance (ANOVA), followed by Scheffè’s post hoc analysis. The null hypothesis (H_0_) was rejected for *p* ≤ 0.05.

## Data Availability

The data that supports the findings of this study are available from the corresponding author upon reasonable request.

## Supplementary Materials

The following supporting information can be downloaded at: https://www.mdpi.com/article/10.3390/ijms23168926/s1.
