# Supplementary material for: Alterations of Mitochondrial Structure in Methamphetamine Toxicity
Source: Int J Mol Sci. 2022 Aug 10;23(16):8926. doi: 10.3390/ijms23168926 (PMC9408775; doi:10.3390/ijms23168926)
Supplement: Supplementary file 1 [file ijms-23-08926-s001.zip › ijms-1825406-supplementary.pdf]

A

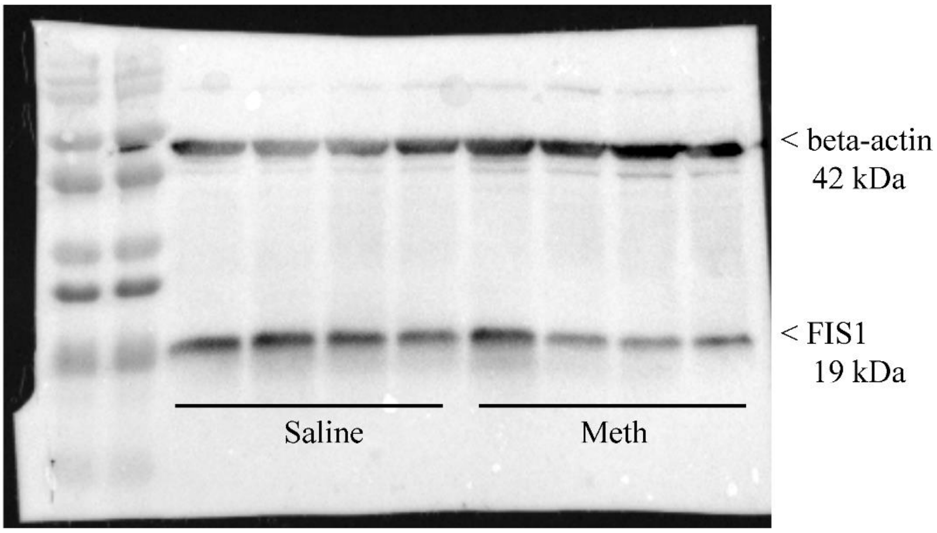

B

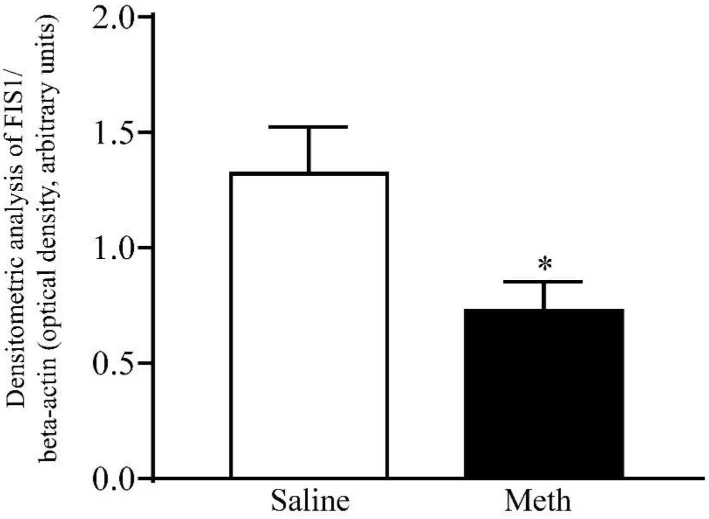

Supplementary Figure S1. METH (100 μM) decreases the fission protein Fis1. (**A**) Representative blots of Fis1 protein. (**B**) Graph reports METH-induced decrease of Fis1 levels. Values are given as the mean±S.E.M. from 4 samples per group. *$p \leq 0.05$ compared with controls.

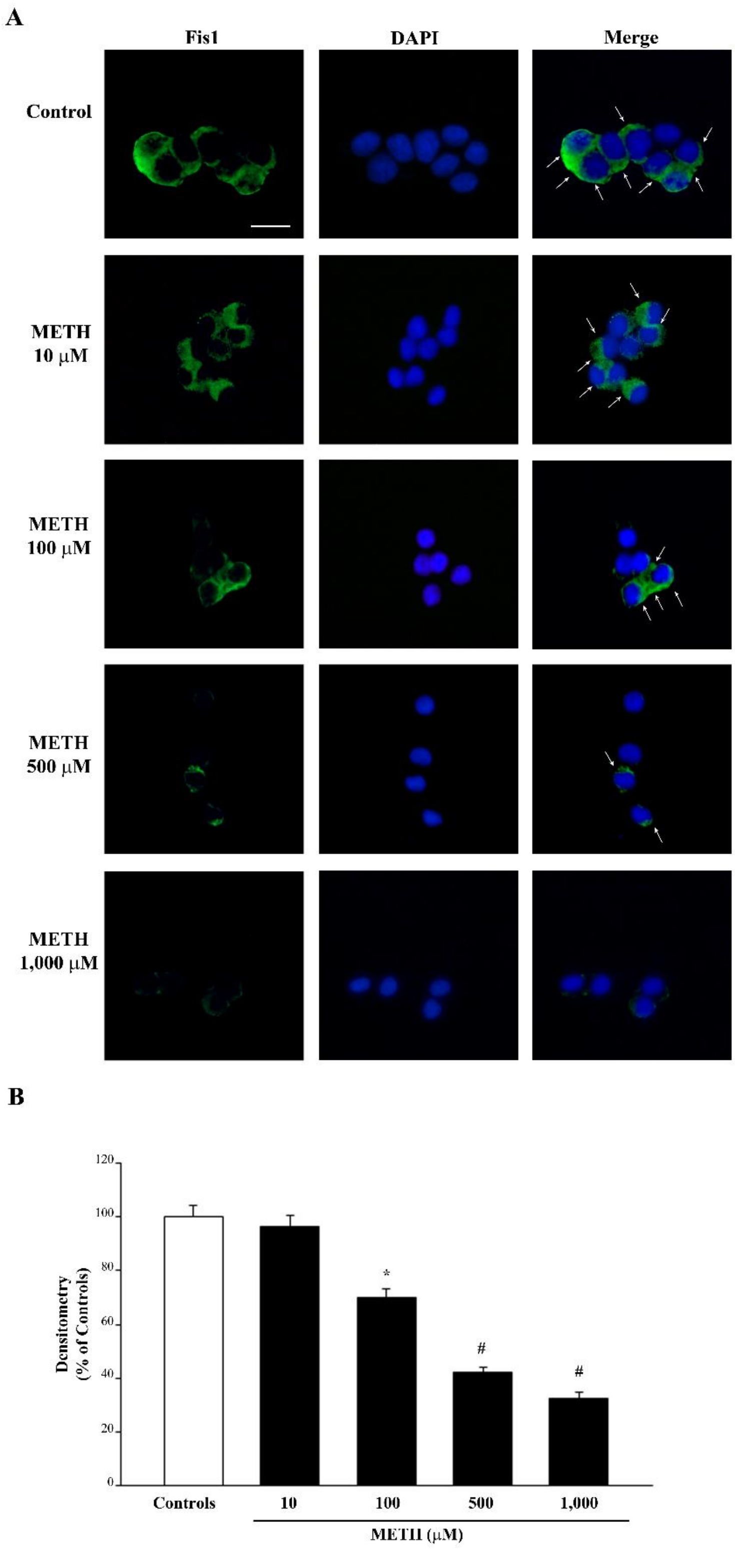

Supplementary Figures S2. METH decreases immunofluorescence for Fis1. (**A**) Representative pictures of the fission marker Fis1 in control and following METH treatment (from 10 μM up to 1,000 μM). Arrows point to intense fluorescent areas. (**B**) The graph reports densitometry of Fis1 immunofluorescence. Values are given as the mean±S.E.M from N=100 cells per group. $^{*}p \leq 0.05$ compared with controls and MTH 10 μM; $^{\#}p \leq 0.05$ compared with controls and METH up to 100 μM. Scale bar=13 μm.

A

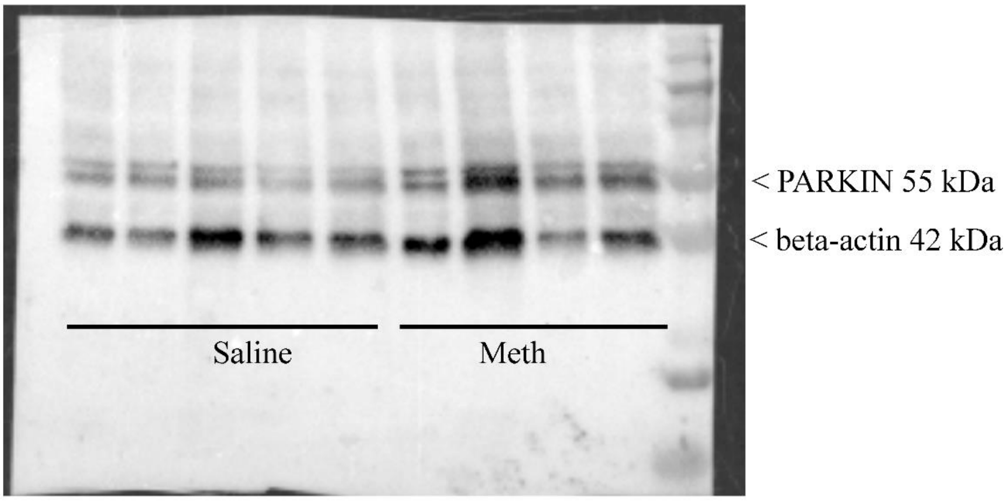

B

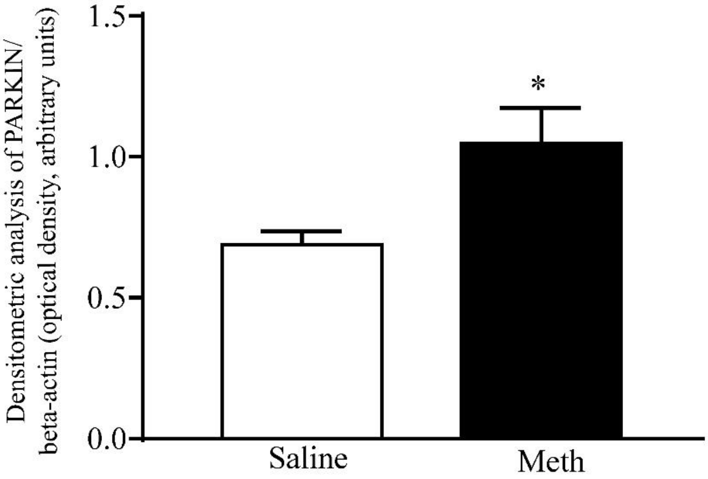

Supplementary Figure S3. METH (100 μM) increases the protein Parkin. (**A**) Representative blots of Parkin. (**B**) Graph reports the METH-induced increase of the levels of Parkin. Values are given as the mean±S.E.M. from at least 4 samples per group. *$p \leq 0.05$ compared with controls.

A

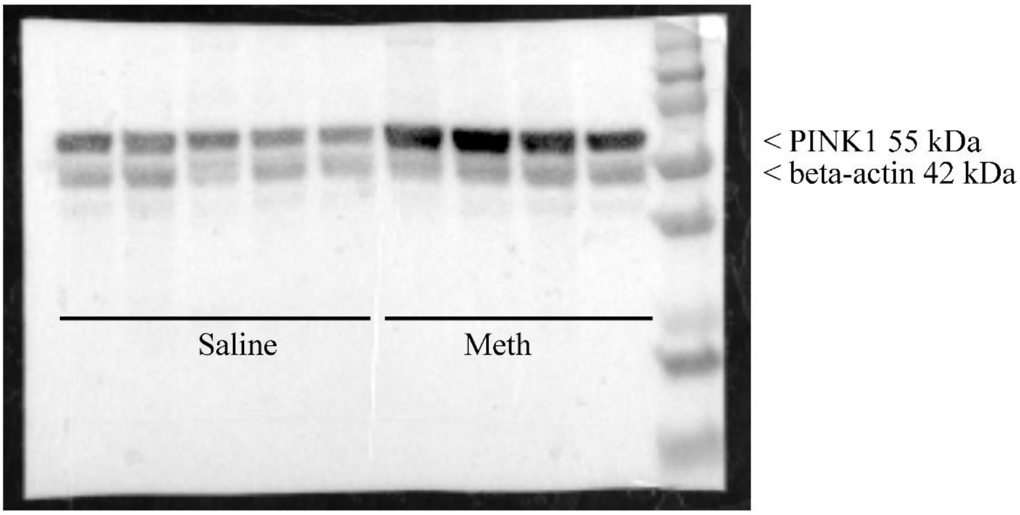

B

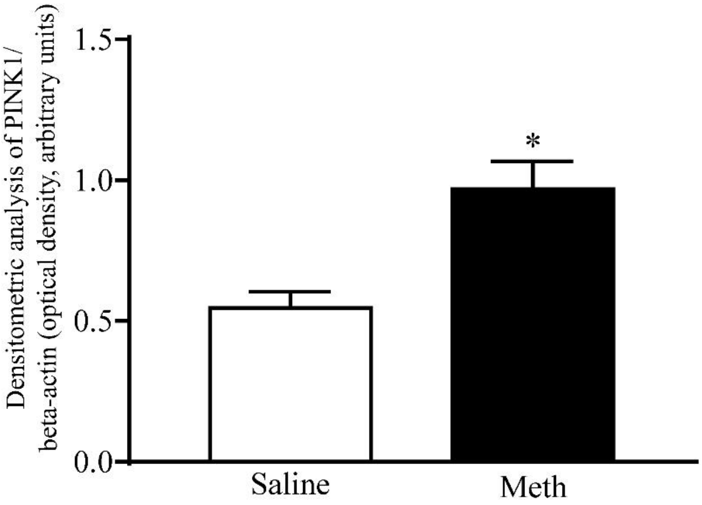

Supplementary Figure S4. METH (100 μM) increases the protein Pink1. (**A**) Representative blots of Pink1. (**B**) Graph reports METH-induced increase of Pink1. Values are given as the mean±S.E.M. from at least 4 samples per group. *$p \leq 0.05$ compared with controls.

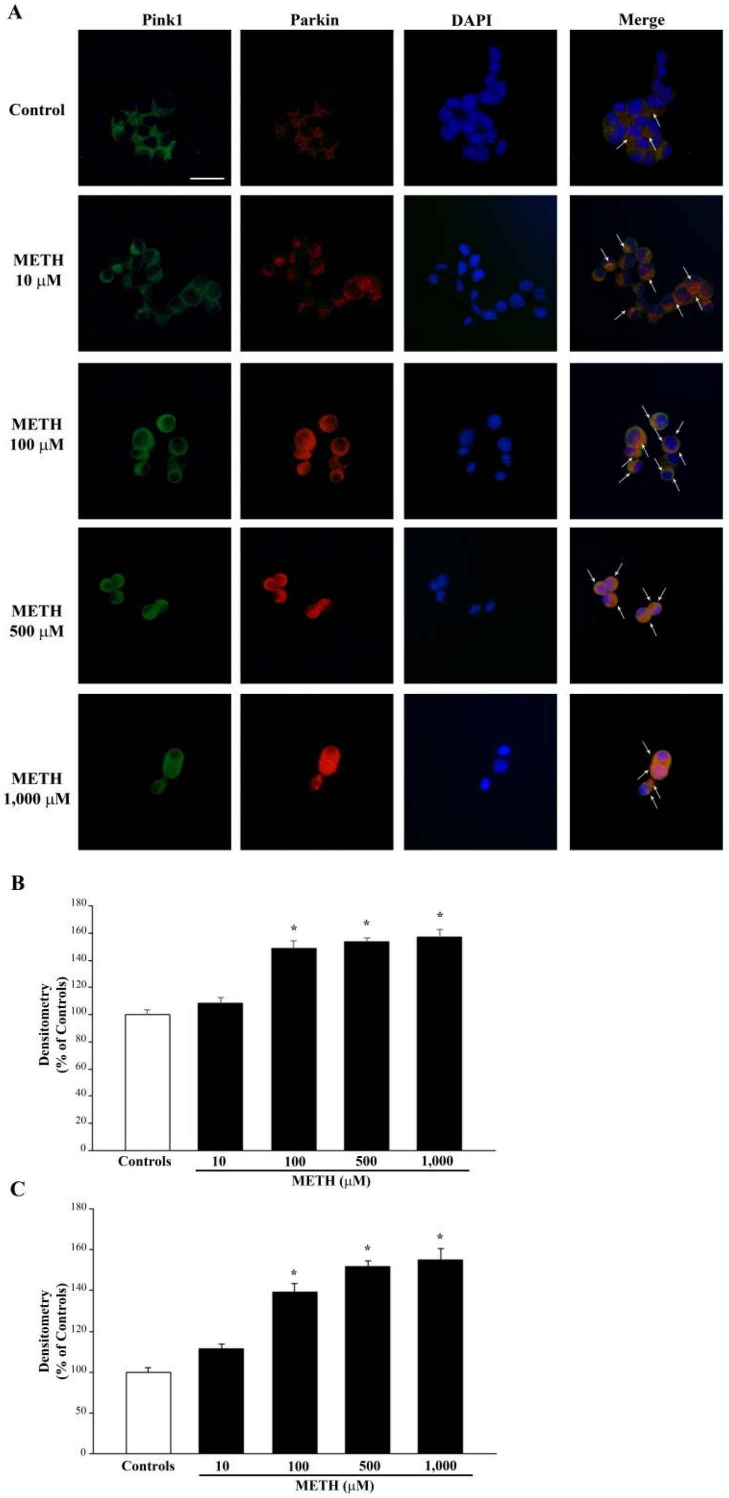

Supplementary Figure S5. METH increases both Pink1 and Parkin immunofluorescence. (**A**) Representative pictures show Pink1 and Parkin immunofluorescence from control and METH-treated cells (from 10 μM up to 1,000 μM). Arrows point to merging (yellow) areas. The graphs report the densitometry of Pink1 immunofluorescence (**B**), and Parkin immunofluorescence (**C**). Data are given as the mean±S.E.M. from N=90 cells per group. **p*≤0.05 compared with controls. Scale bar=17μm.

A

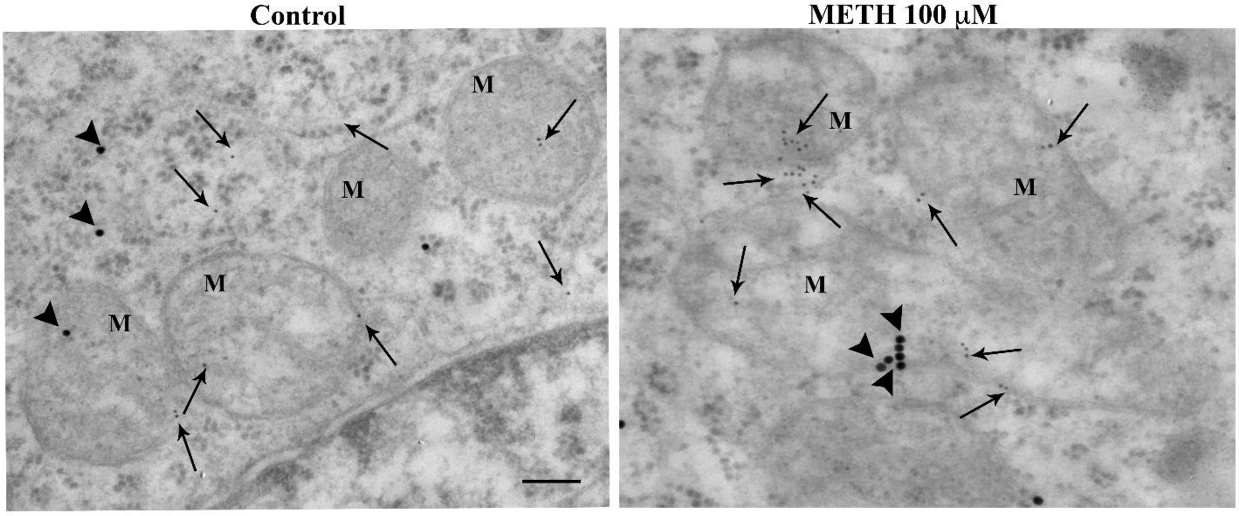

B

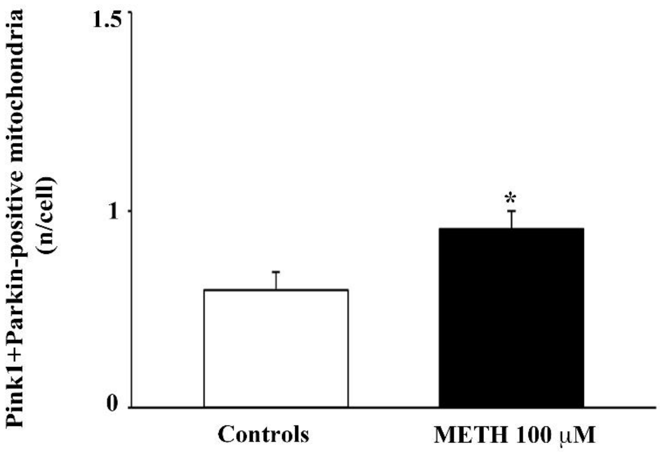

Supplementary Figure S6. METH increases the mitochondrial co-localization of Pink1 and Parkin. (**A)** Representative TEM micrographs showing Pink1- and Parkin- immunogold particles in control and METH 100 μM-treated cells. Arrows point to Parkin immunogold particles (10 nm), while arrowheads point to Pink1 immunogold particles (20 nm) within mitochondria. M=mitochondria. (**B**) Graph report the number of Pink1+Parkin-positive mitochondria. Values are given as the mean±S.E.M. counted in 50 cells per group. *$p \leq 0.05$ compared with controls. Scale bar=170 μm.

A

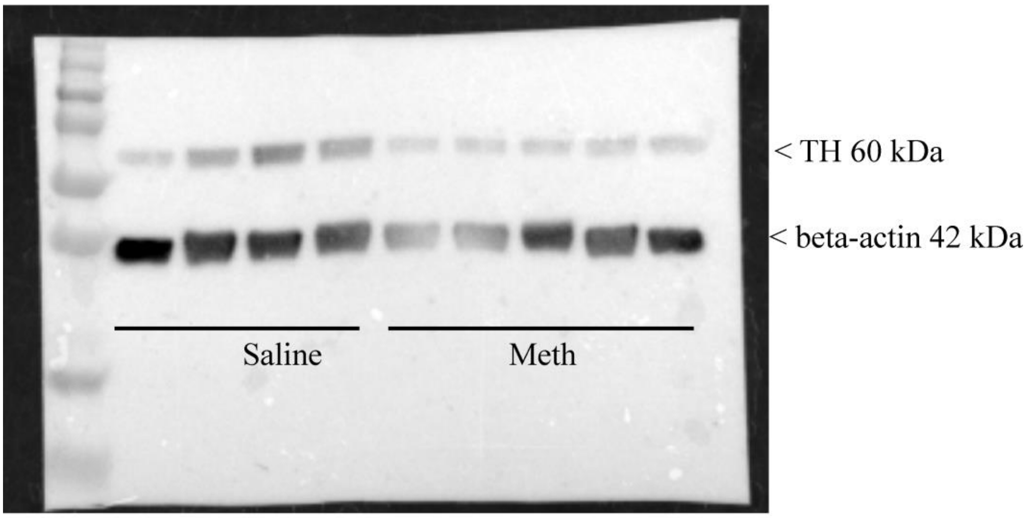

B

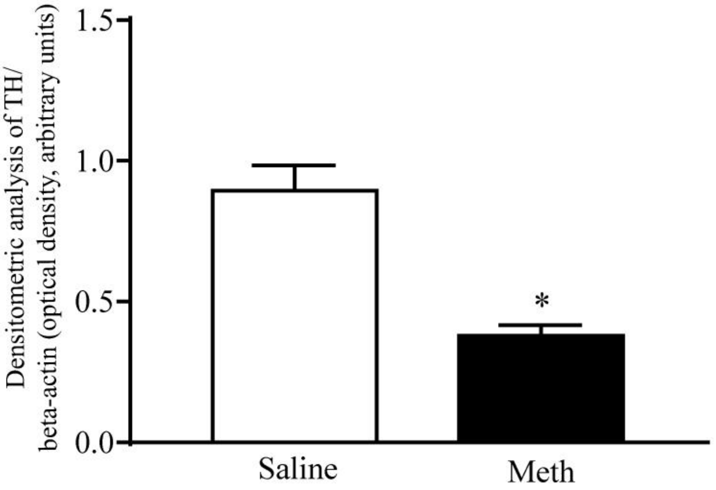

Supplementary Figure S7. METH (100 μM) decreases tyrosine-hydroxylase (TH). (**A**) Representative blots of TH. (**B**) Graph reports METH-induced decrease of the levels of TH. Values are given as the mean±S.E.M. from at least 4 samples per group. **p*≤0.05 compared with controls.
